# An Overview of the Systematic Reviews About the Efficacy of Fluvoxamine on Depression

**DOI:** 10.3390/ph18050711

**Published:** 2025-05-12

**Authors:** Luiz Henrique Junqueira Dieckmann, Michel Haddad, Thiago Wendt Viola, Franciele Franco Scarante, Naielly Rodrigues da Silva, Jair de Jesus Mari

**Affiliations:** 1 Department of Psychiatry, Brazilian Clinical Research Institute, São Paulo 01404-000, SP, Brazil; dieckmann@grupobipp.com.br (L.H.J.D.); haddad@grupobipp.com.br (M.H.); franfscarante@grupobipp.com.br (F.F.S.); naiellyrodrigues@grupobipp.com.br (N.R.d.S.); 2 Department of Psychiatry, Universidade Federal de São Paulo, São Paulo 04017-030, SP, Brazil; 3 School of Medicine, Brain Institute of Rio Grande do Sul, Pontifical Catholic University of Rio Grande do Sul (PUCRS), Porto Alegre 90619-900, RS, Brazil; thiago.wendt@pucrs.br

**Keywords:** fluvoxamine, depression, antidepressant, systematic reviews, meta-analyses

## Abstract

**Background:** Depression is one of the leading causes of disability worldwide. Among pharmacological treatments, fluvoxamine—an early SSRI with a distinct pharmacological profile—has been recently reappraised for its broader clinical relevance. Objective: To assess the efficacy of fluvoxamine in the treatment of depression compared to placebo and other antidepressants through a comprehensive overview of systematic reviews and meta-analyses. Methods: A systematic search was conducted in MEDLINE and the Cochrane Central Register of Controlled Trials, including systematic reviews and meta-analyses of randomized controlled trials evaluating fluvoxamine’s efficacy. Reviews were eligible if they included adults diagnosed with depressive disorders based on the DSM or ICD criteria. Reviews focusing on other psychiatric disorders, comorbidities, tolerability, or economic evaluations were excluded. Data extraction included effect size measures and methodological quality assessments using the AMSTAR-2 tool. Results were synthesized by comparing fluvoxamine to placebo, tricyclic antidepressants (TCAs), selective serotonin reuptake inhibitors (SSRIs), serotonin-norepinephrine reuptake inhibitors (SNRIs), and other antidepressants. **Results:** A total of 74 reviews were identified, of which 14 systematic reviews met the inclusion criteria after screening and full-text analysis. These reviews, published between 1994 and 2021, predominantly involved nine pairwise meta-analyses and five network meta-analyses, comparing fluvoxamine with placebo and various antidepressants. Fluvoxamine demonstrated consistent superiority over placebo in achieving treatment response and remission outcomes. Comparisons with imipramine, clomipramine, amitriptyline, dothiepin, paroxetine, fluoxetine, citalopram, mianserin, nortriptyline, and moclobemide generally revealed no significant differences in efficacy. However, some reviews indicated that venlafaxine and mirtazapine were superior to fluvoxamine in certain outcomes, while fluvoxamine demonstrated greater efficacy than desipramine in one review. Sertraline and milnacipran showed mixed or review-quality-dependent results, with one low-quality review favoring milnacipran. Most reviews assessed outcomes over a median follow-up of six weeks using standardized depression rating scales. **Conclusions:** Fluvoxamine is a robust and effective antidepressant, demonstrating consistent efficacy comparable to other antidepressants and superior to placebo. While no single antidepressant was universally superior, fluvoxamine’s unique pharmacological profile and favourable safety characteristics support its clinical utility. Further research is needed to explore its role in personalized treatment strategies and emerging therapeutic contexts, such as comorbid anxiety and post-traumatic stress disorder.

## 1. Introduction

Depression is one of the leading causes of disability worldwide [1]. In countries like Brazil, the USA, and France—nations marked by significant social disparities—nearly one in five people will experience a clinically relevant depressive disorder at some point in their lives, and only a minority of people with major depression receive any treatment [2]. Several factors contribute to the risk of developing depression, including biological factors such as genetics, neurotransmitter imbalances, and medical conditions [3].

The incidence of depression has been increasing over time [1]. The point prevalence of elevated depressive symptoms among adolescents increased from 24% to 37% by comparing data from 2001 and 2020, with the COVID-19 pandemic further exacerbating this trend [4]. Females are nearly twice as likely as males to experience depression, according to nationally representative samples [5]. Cannabis use in adolescence has been associated with an increased risk of developing depression, while depression subsequently elevates the likelihood of problematic cannabis use, suggesting the presence of a reinforcing bidirectional cycle [6]. The prevalence of depression also varies across the lifespan, with late life being particularly affected by the comorbidity of chronic illnesses, mobility impairments, and psychosocial stressors in this vulnerable population [7,8]. The rising prevalence of depression among adolescents highlights the influence of gender, dysfunctional coping mechanisms, the introduction of new digital technologies, and socio-environmental stressors in shaping depressive outcomes and increasing the risk of suicide during this developmental stage [5,6].

Depression comprises distinct subtypes that differ in symptomatology, disease course, and etiology [9,10]. Neurobiological hallmarks of depression, such as hypothalamic-pituitary-adrenal (HPA) axis dysregulation and changes in inflammatory and metabolic mediators, vary between melancholic and atypical depression [11]. Highlighting the heterogeneity in stress responses across these clinical presentations, it was identified distinct variations in both objective and subjective stress measures among individuals with different subtypes of depression [12]. More recent approaches suggest the use of neuroimaging to identify biotypes of depression distinguished by their pattern of circuitry dysfunction [13] or by the pattern of alteration of neurotransmission systems or other biological markers [9]. This complexity and heterogeneous nature of depression challenges both the proper diagnosis and the appropriate treatment selection.

The pharmacotherapy of depression was revolutionized in the 1950s and 1960s with the serendipitous discovery of drugs with mood-improving properties [14]. The characterization of those newly discovered antidepressants as modulators of the monoaminergic neurotransmission led the pharmaceutical industries to design new antidepressants that acted by selectively targeting the monoaminergic system, with a particular focus on selective serotonin reuptake inhibitors (SSRIs) [14]. Fluvoxamine was one of the first SSRIs to be launched for the treatment of depression [15]. It was developed by Kali-Duphar (Solvay Pharmaceuticals, now Abbott Laboratories) and has been available in the market since 1983 [15]. Fluvoxamine is a monocyclic SSRI from the class of 2-aminoethyloximethers of aralkylketones [16,17]. It exhibits high selectivity for the serotonergic transporter (Ki = 6.2 nM) and low affinity for the noradrenergic transporter (Ki = 1100 nM) [18]. Additionally, fluvoxamine acts as a sigma-1 receptor agonist, a property that distinguishes it from other SSRIs and may contribute to its therapeutic effects [19,20,21].

Growing evidence implicates the sigma-1 receptor in the pathophysiology of neuropsychiatric conditions, as depression, with cognitive and inflammatory components [20,22]. Its antidepressant effects appear to involve multiple mechanisms, including calcium homeostasis, modulation of neurotransmitter systems, enhancement of brain-derived neurotrophic factor (BDNF), and others [22,23]. The sigma-1 receptor also plays a critical role in systemic inflammation and cytokine regulation [22,23]. Notably, fluvoxamine has recently attracted renewed interest for its potential benefits in patients with COVID-19 [24,25,26]. Thus, the varying affinities of antidepressants for the sigma-1 receptor may, in part, explain the heterogeneity observed in their clinical effects.

Despite the availability of numerous pharmacological agents approved for the treatment of depression, a substantial proportion of patients fail to achieve adequate symptom remission after multiple interventions, leading to what is defined as treatment-resistant depression (TRD) [27]. This clinical challenge not only leads to persistent functional impairment and increased suicide risk but also places a significant burden on healthcare systems [28,29]. Such outcomes underscore the limitations of current monoaminergic-based approaches and highlight the pressing need for alternative strategies that address the complex neurobiology of depression, including neuroinflammatory processes, neuroplasticity disruptions, and metabolic dysfunction [30].

Although fluvoxamine is widely recognized for its indication in the treatment of obsessive-compulsive disorder (OCD) in adults and adolescents, it is also effective for major depressive disorder (MDD) [31,32,33]. Off-label uses include anxiety disorders, post-traumatic stress disorder (PTSD), and, more recently, COVID-19 [25,31,34,35]. Fluvoxamine is contraindicated in patients with hypersensitivity to the drug and in those receiving monoamine oxidase inhibitors (MAOIs) or thioridazine, alosetron, or pimozide due to the risk of severe serotonergic and cardiovascular events [33]. It is rapidly absorbed after oral administration with oral bioavailability of approximately 50% due to first-pass hepatic metabolism [36,37]. Fluvoxamine has a half-life of 15–20 h and undergoes extensive hepatic metabolism via CYP1A2 and CYP2C19 isoenzymes [37]. Due to its strong CYP inhibition, clinically relevant interactions may occur with some drugs as propranolol, theophylline, warfarin, and certain tricyclic antidepressants and benzodiazepines [37]. Common adverse effects reported with the use of fluvoxamine include nausea, insomnia, somnolence, dizziness, and gastrointestinal symptoms [33]. During pregnancy, its use should be reserved for cases in which potential maternal benefits outweigh fetal risks, given the limited data in humans [33,38].

Given the clinical complexity and biological heterogeneity of major depressive disorder, alongside the limitations of monoaminergic-based treatments and the growing interest in alternative mechanisms such as sigma-1 receptor modulation, a comprehensive synthesis of the existing evidence on fluvoxamine is warranted to elucidate its comparative efficacy within the SSRIs. This manuscript aims to present an overview of systematic reviews and meta-analyses evaluating the efficacy of fluvoxamine in the treatment of depression, compared to placebo and other antidepressants, with the goal of elucidating its therapeutic role within the contemporary landscape of antidepressant pharmacotherapy.

## 2. Results

**Figure 1:**
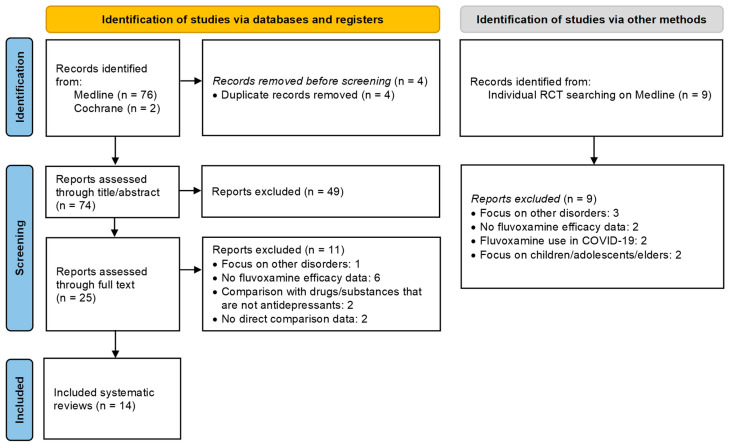
PRISMA 2020 flow diagram for articles included in the manuscript. Source: [58].

After removing duplicates, 74 unique records were screened by title and abstract, resulting in the exclusion of 49 records. Out of the 25 articles still eligible for inclusion, full-text analysis led to the exclusion of 11, and the remaining 14 reviews were found suitable for inclusion [39,40,41,42,43,44,45,46,47,48,49,50,51,52] (Figure 1). The reasons for the exclusion of the studies are highlighted in the flowchart (Figure 1). The publication dates of the included reviews ranged from 1994 to 2023, with the most recent being conducted by Kishi et al. (2023) [52] using a search cutoff date of 22 May 2022 [52]. Our additional search identified five potential RCTs [53,54,55,56,57] published after the study by Kishi et al. (2023) [52], but none were eligible for inclusion.

The included reviews compared the efficacy of fluvoxamine against placebo, imipramine, clomipramine, amitriptyline, dothiepin, desipramine, paroxetine, sertraline, fluoxetine, citalopram, milnacipran, venlafaxine, mirtazapine, mianserin, nortriptyline, and moclobemide. Most treatment outcomes were measured as response rates using either the Hamilton Depression Rating Scale or the Montgomery–Åsberg Depression Rating Scale. The most common follow-up period was 6 weeks, although durations in the RCTs ranged from 4 to 52 weeks, with the longest follow-up associated with remission outcomes. In most included studies, fluvoxamine was administered orally at doses ranging from 50 to 300 mg per day, consistent with standard clinical practice.

The methodological quality assessment of the included systematic reviews, conducted using AMSTAR-2, revealed that six studies were rated as high quality [39,40,46,47,49,52], five as moderate quality [44,45,48,50,51], and three as low quality [41,42,43]. Item-by-item ratings are presented in Table S3, along with the correlation plot (Figure S1). A correlation analysis of the quantitative scores (ranging from 3 to 16 points) and the year of publication revealed a strong association (Pearson’s r = 0.79, *p* < 0.0001) (Figure S1). As expected, this indicates that more recent studies demonstrated higher methodological rigor (Figure S1).

**Figure 2:**
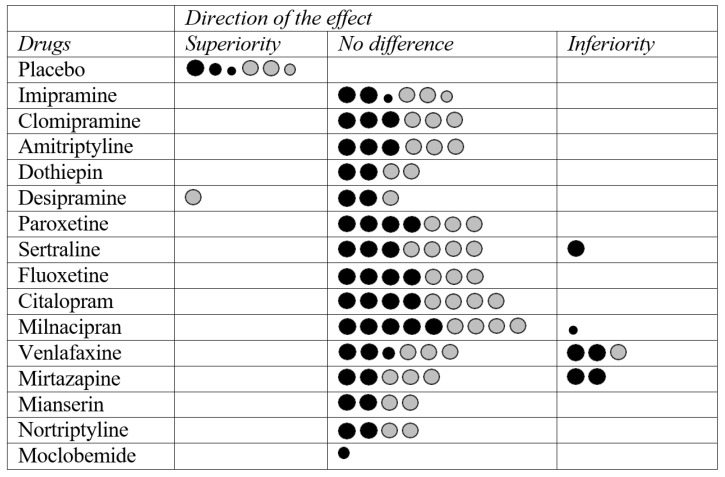
Schematic representation of the number of reviews providing evidence on fluvoxamine’s efficacy in depression treatment. Dot size was proportional to the AMSTAR-2 classification score of the review (high, moderate, or low). Black dots represent efficacy data estimated based on treatment response, while grey dots represent efficacy data estimated based on remission.

Figure 2 presents a graphical representation of the number of reviews assessing fluvoxamine’s efficacy compared to other drugs, considering response and remission, while also accounting for the AMSTAR-2 score and the effect (e.g., superiority, inferiority, or no difference). This graphical representation highlights that milnacipran, citalopram, sertraline, and venlafaxine were the drugs with the highest number of reviews analyzing their efficacy against fluvoxamine (Figure 2). Table 1 summarizes the characteristics of high-quality reviews based on the AMSTAR-2 assessment and head-to-head comparisons of fluvoxamine’s efficacy assessed by response to treatment, while Table 2 provides the same information for fluvoxamine’s efficacy assessed by remission. The summaries include the type of review, number of included RCTs, sample size, follow-up duration, blinding method, measure of efficacy, statistical summary estimates, and main findings. Most of the head-to-head comparisons showed very high CCA percentages, indicating a frequent overlap of RCTs analyzed across the included reviews (Table S4). Data for all included reviews are provided in Table S4.

### 2.1. Fluvoxamine vs. Placebo

Evidence of fluvoxamine’s efficacy against placebo included one high-quality review [49], one moderate-quality review [50], and one low-quality review [41] that were considered for the response to treatment analysis, while two high-quality reviews [49,52] and one moderate-quality review [50] were considered for the remission analysis. Focusing specifically on high-quality reviews, Cipriani and collaborators (2018) included 14 RCTs [59,60,61,62,63,64,65,66,67,68,69,70,71,72] totaling 1799 patients with a follow-up time ranging between 4–6 weeks to demonstrate the superiority of fluvoxamine over placebo considering treatment response and remission outcomes [49]. In terms of remission, a review by Kishi and collaborators (2023) [52] included a single RCT [73] with 204 patients in a 52-week follow-up study that also demonstrated fluvoxamine’s superiority over placebo.

### 2.2. Fluvoxamine vs. Tricyclic Antidepressants (TCAs)

Regarding fluvoxamine versus imipramine, two high-quality reviews [39,40] were considered for both the treatment response and the remission analysis. Omori and collaborators (2009) included 6 RCTs [60,62,68,69,74,75] totaling 282 patients with a follow-up time of 6 weeks [39], and Omori and collaborators (2010) included 7 RCTs [62,64,65,66,68,69,74] totaling 422 patients with a follow-up time of 6 weeks [40]. Both reviews demonstrated no significant differences between the drugs in terms of treatment response and remission.

For fluvoxamine versus clomipramine, three high-quality reviews [39,40,49] were considered for both the response to treatment and the remission analysis. In both reviews conducted by Omori and collaborators (2009 and 2010), 1 RCT [76] was included, totaling 86 patients with a follow-up time of 8 weeks for the response [40] and remission outcomes [39]. Also, Omori and collaborators (2010) included 1 additional RCT [77] for the response outcome analysis totaling 159 patients with a follow-up time ranging from 6–8 weeks [40]. Cipriani and collaborators (2018) included 2 RCTs [77,78] totaling 83 patients with a follow-up time ranging from 4–6 weeks for response and remission outcomes [49]. All three reviews demonstrated no significant differences between drugs in both efficacy outcomes considered (treatment response and remission).

Concerning fluvoxamine versus amitriptyline, three high-quality reviews [39,40,49] were considered for both the treatment response and the remission analysis. Both reviews conducted by Omori and collaborators (2009, 2010) included the same 4 RCTs [79,80,81,82] totaling 185 patients with a follow-up time ranging from 6–7 weeks [39,40], while Cipriani and collaborators (2018) included 3 RCTs [80,82,83] totaling 337 patients with a follow-up time ranging from 4–7 weeks [49]. These reviews demonstrated no significant differences between the drugs in terms of treatment response and remission. Regarding fluvoxamine versus dothiepin, both high-quality reviews conducted by Omori and collaborators (2009, 2010) included the same 2 RCTs [84,85] totaling 125 patients with a follow-up time of 6 weeks [39,40]. The reviews showed that treatment response and remission did not differ between the two drugs.

For fluvoxamine versus desipramine, the two high-quality reviews conducted by Omori and collaborators (2009, 2010) included the same single RCT [86] totaling 47 patients with a follow-up time of 10 weeks [39,40]. Despite there being no significant differences between the drugs in the treatment response analysis, fluvoxamine showed superiority over desipramine in one [40] out of two reviews that included data on remission [39,40].

In the comparison between fluvoxamine and nortriptyline, two reviews conducted by Omori and colleagues (2009, 2010) analyzed the same single RCT [87], encompassing 74 patients with a follow-up period of 8 weeks [39,40]. These reviews found no significant differences between the two drugs [39,40]. Further analyses comparing fluvoxamine with unspecified tricyclic antidepressants included two high-quality reviews [39,40]. One of these reviews [40] included 16 RCTs [62,64,65,68,69,74,76,77,79,80,81,82,84,85,86,87] encompassing 965 patients with a follow-up time ranging from 6–10 weeks. The second high-quality review [39] included 16 RCTs [60,62,68,69,74,75,76,77,79,80,81,82,84,85,86,87] encompassing 872 patients with a follow-up time ranging from 6–10 weeks. Both reviews indicated no significant differences between fluvoxamine and tricyclic antidepressants on response or remission outcomes [39,40].

### 2.3. Fluvoxamine vs. SSRIs

Regarding fluvoxamine versus paroxetine, four high-quality reviews [39,40,46,49] provided evidence of fluvoxamine’s efficacy assessed by treatment response, and three high-quality reviews [39,40,49] provided evidence considering remission. The reviews conducted by Omori and collaborators (2009, 2010) and Cipriani (2009) included the same 3 RCTs [88,89,90] totaling 281 patients with a follow-up time ranging from 6–7 weeks [39,40]. The review conducted by Cipriani and collaborators (2018) included 2 RCTs [88,90] totaling 180 patients, with a follow-up time ranging from 6–7 weeks [49]. All reviews did not find significant differences between the drugs in terms of response to treatment and remission.

Regarding fluvoxamine versus sertraline, four high-quality reviews [39,40,46,49] provided evidence of fluvoxamine’s efficacy assessed by treatment response, and four high-quality reviews [39,40,48,49] provided evidence considering remission. The reviews conducted by Omori and collaborators (2009, 2010) and Cipriani and collaborators (2009, 2018) included the same 2 RCTs [91,92] totaling 185 patients with a follow-up time of 7 weeks [39,40,46,49]. The review conducted by Ramsberg and collaborators (2012) [48] included only one of the RCTs included in the previous reviews [92]. Except for one review that indicated sertraline’s superiority over fluvoxamine on response outcome [46], the remaining reviews demonstrated no significant differences between the drugs in any of the efficacy outcomes [39,40,48,49].

Regarding fluvoxamine versus fluoxetine, four reviews [39,40,46,56] provided evidence of fluvoxamine’s efficacy assessed by treatment response, and three reviews [39,40,49] provided evidence considering remission. All of these high-quality reviews included the same 2 RCTs [91,92] totaling 284 patients, with a follow-up time ranging from 6–7 weeks [39,40,48,49]. The reviews found no significant differences between the drugs in terms of treatment response and remission.

Regarding fluvoxamine versus citalopram, four reviews [39,40,46,49] provided evidence of fluvoxamine’s efficacy assessed by treatment response, and four reviews [39,40,48,49] provided evidence considering remission. All of these high-quality reviews included the same single RCT [93] with 217 patients, with a follow-up time of 6 weeks [39,40,46,49]. No significant differences between the drugs in terms of treatment response and remission were observed in all of the reviews.

Further analyses comparing fluvoxamine with unspecified SSRIs involved one high-quality review [40] that included 8 RCTs [88,89,90,91,92,93,94,95] encompassing 967 patients with a follow-up time ranging from 6–7 weeks. There were no significant differences of fluvoxamine with unspecified SSRIs on response or remission outcomes.

### 2.4. Fluvoxamine vs. SNRIs

Considering fluvoxamine versus milnacipran, five high-quality [39,40,46,47,49] and one low-quality review [43] provided evidence of fluvoxamine’s efficacy assessed by treatment response, and three high-quality [39,40,47] and one low-quality review [43] provided evidence considering remission. The high-quality reviews conducted by Omori and collaborators (2009, 2010), Cipriani and collaborators (2009), and Nakagawa and collaborators (2009) included the same single RCT [96] with 113 patients, with a follow-up time of 6 weeks [39,40,46,47]. Cipriani and collaborators (2018) included an additional RCT [97] totaling 239 patients, with a follow-up time ranging from 4–6 weeks [49]. All of these high-quality reviews indicated no significant differences between the drugs in terms of treatment response and remission. However, the low-quality review conducted by Lopez-Ibor et al. (1996) indicated superiority of milnacipran over fluvoxamine regarding treatment response [43].

Regarding fluvoxamine versus venlafaxine, four high-quality [39,40,46,49] and one moderate-quality review [45] provided evidence of fluvoxamine’s efficacy assessed by treatment response, and four high-quality reviews [39,40,48,49] provided evidence considering remission. Cipriani et al. (2009) [46], Omori et al. (2009) [39], Nemeroff et al. (2008) [45], and Ramsberg et al. (2012) [48] included a single RCT [98], but the number of patients varied from 71 to 111, with a follow-up time of 6 weeks. The second review by Cipriani and collaborators (2018) included 2 studies [98] an unpublished clinical trial [49]. Also, Omori and collaborators, in their second review (2010) [40], included two separate studies that were considered to originate from the same dataset reported by Hackett et al., 1998 [98]. Two [40,46] of the four high-quality reviews [39,40,46,49] indicated that venlafaxine is superior to fluvoxamine in terms of treatment response, while one review [48] indicated similar superiority of venlafaxine in the remission outcome.

The comparison of fluvoxamine with unspecified SNRIs included 2 high-quality reviews [39,40], which included the same 2 RCTs [96] encompassing a total of 967 patients with a follow-up time ranging from 6–7 weeks. The reviews showed no significant differences in fluvoxamine compared with unspecified SNRIs on response or remission outcomes, except for the review by Omori et al. (2010), which indicated that, in general, SNRIs are superior to fluvoxamine in treatment response outcomes [40].

### 2.5. Fluvoxamine vs. Other Antidepressants

Regarding fluvoxamine versus mirtazapine, four high-quality reviews [39,40,46,49] provided evidence of fluvoxamine’s efficacy assessed by treatment response, and three high-quality reviews [39,40,49] provided evidence considering remission. The reviews conducted by Omori and collaborators (2009, 2010) and Cipriani and collaborators (2009) included the same RCT [99] totaling 412 patients, all with a follow-up time of 6 weeks [39,40,46]. Cipriani et al. (2018) included 1 RCTs [100] totaling 412 patients with a follow-up time of 6 weeks [49]. Two [39,49] of these four reviews [39,40,46,49] indicated that mirtazapine is superior to fluvoxamine in terms of response to treatment, while no differences between the drugs were observed, considering the remission outcome.

Considering mianserin, the two high-quality reviews conducted by Omori and collaborators (2009, 2010) [39,40] included the same 2 RCTs [101,102] with 125 patients, with a 6-week follow-up period for both response and remission analyses. No significant differences were found between mianserin and fluvoxamine.

For the comparison with moclobemide, only the moderate-quality review [51] included two RCTs, but no information on these trials included in the analysis was available. This comparison indicated no significant differences between drugs in terms of treatment response.

## 3. Discussion

The goal of this overview was to evaluate fluvoxamine’s efficacy in comparison to placebo or other antidepressants. The findings highlight fluvoxamine’s consistent superiority over placebo, both in terms of treatment response and remission. This reinforces the drug’s clinical utility as a serotonergic antidepressant with proven efficacy. However, when compared to other antidepressants, TCAs, SSRIs, SNRIs, and other newer agents, the evidence paints a more nuanced picture, with no substantial differences in efficacy for most comparisons.

The superiority of fluvoxamine over placebo was consistently demonstrated across both treatment response and remission outcomes (Table 1 and Table 2). High-quality reviews, particularly the study performed by Cipriani et al. (2018), provided robust evidence for fluvoxamine’s effectiveness, supported by data from numerous RCTs [49]. These findings are critical in confirming fluvoxamine’s role in clinical practice as a reliable option for treatment of depression.

The efficacy of fluvoxamine in treating depression compared to placebo is consistent with robust evidence supporting the role of serotonin-targeting antidepressants in managing depression symptoms [103]. Hieronymus and colleagues (2016) performed a comprehensive post hoc analysis of 18 industry-sponsored placebo-controlled trials and described that SSRIs are consistent in decreasing depressed mood in comparison to placebo [103]. Furthermore, a recent study used network estimation techniques to show that SSRIs are primarily effective in reducing affective symptoms, and these primary effects are related to the secondary improvement of cognitive and somatic symptoms [104].

Despite its superiority over placebo, fluvoxamine exhibited comparable efficacy to SSRIs like paroxetine, sertraline, fluoxetine, and citalopram [40]. This parity is consistent with previous meta-analyses of SSRIs, which generally report minor or no significant differences in efficacy across the class [40]. Cipriani and colleagues (2009) described in a multiple-comparison meta-analysis that sertraline was more efficacious than fluvoxamine [46]. However, the study highlights that the benefit of sertraline over fluvoxamine is not clear since the credibility interval for OR was only slightly more than 1 [46].

When compared to TCAs like imipramine, clomipramine, and amitriptyline, fluvoxamine generally demonstrated similar efficacy. The absence of significant differences highlights the equivalent therapeutic potential of fluvoxamine and TCAs, albeit with differing safety and tolerability profiles. This equivalence may be particularly relevant in tailoring treatment to patient-specific factors, such as comorbidities or tolerability preferences. Although based on limited data, the evidence suggesting fluvoxamine’s superiority over desipramine in terms of remission outcomes [40] points to potential benefits in certain subpopulations. Given fluvoxamine’s more favorable tolerability profile, lower anticholinergic burden, and sigma-1 receptor agonism, it may offer advantages in patients for whom desipramine is less suitable—such as older adults or individuals with comorbid anxiety or increased cardiovascular risk [31,105]. Nevertheless, further high-quality comparative studies are needed to confirm this potential advantage and to better define the specific clinical contexts in which it may be most relevant.

The evidence comparing fluvoxamine to SNRIs like venlafaxine and milnacipran indicates largely similar efficacy, though some reviews noted venlafaxine’s superiority in response rates [39,40]. This aligns with previous findings suggesting that SNRIs may have marginally greater efficacy in severe depression due to their dual-action mechanism targeting both serotonin and norepinephrine system [106,107,108].

The superiority of SNRIs in some cases highlights their potential as alternatives for patients with partial responses to SSRIs [106,107,108]. However, tolerability and side effect profiles must be carefully weighed when considering SNRIs over fluvoxamine, particularly considering fluvoxamine’s favorable safety profile and its action as a sigma-1 receptor agonist, which may confer additional therapeutic benefits [21].

The differences between serotonergic antidepressants like fluvoxamine and noradrenergic antidepressants might be related to other clinical features. Studies have shown that there is a gender effect when it comes to response to noradrenergic and serotonergic antidepressants [109,110]. Premenopausal women show a better response to serotonergic, while some studies provide evidence that males might benefit more from noradrenergic-acting antidepressants [109,110]. Naito and colleagues (2007) investigated gender differences in the response to fluvoxamine and milnacipran and found that fluvoxamine was more effective in younger women than in older women and men, while the effect of milnacipran did not seem to differ between genders [111]. These findings, although preliminary, may have clinical implications for tailoring antidepressant choice based on patient sex and age. Fluvoxamine could be considered a preferential option in younger female patients, while milnacipran might offer a more uniform response profile across different demographic groups. Further studies are warranted to confirm and expand upon these potential sex- and age-specific treatment effects.

Fluvoxamine’s comparisons with other antidepressants, such as mirtazapine, mianserin, and moclobemide, yielded no significant differences in most analyses. However, the observed superiority of mirtazapine in some reviews may reflect its distinct pharmacodynamic properties, such as enhanced noradrenergic and serotonergic activity, which could be beneficial for specific patient populations [112]. In a limited number of comparisons, mirtazapine showed superior efficacy to fluvoxamine in certain outcomes. While these findings are not consistent across all studies, they may carry important clinical implications when considering the pharmacological distinctions and therapeutic profiles of both agents.

Mirtazapine, as a noradrenergic and specific serotonergic antidepressant, is known for its sedative properties, appetite stimulation, and lower risk of sexual dysfunction [113]. These characteristics suggest it may be particularly beneficial for patients with depression accompanied by insomnia, anorexia or significant weight loss, or marked anxiety symptoms [113]. In contrast, fluvoxamine may offer more targeted benefits in patients with obsessive-compulsive features or comorbid anxiety [31]. These nuanced distinctions highlight the relevance of tailoring antidepressant selection to patient-specific symptom clusters and comorbidities. While the apparent superiority of mirtazapine in some analyses may inform clinical decision-making in selected populations, further direct comparative studies are warranted to confirm these differences and to support evidence-based personalization of treatment.

The lack of differences between fluvoxamine and moclobemide in limited studies points to the need for further research on monoamine oxidase inhibitors (MAOIs) and their niche utility in treatment-resistant depression [114]. Although MAOIs are rarely used due to safety concerns, their efficacy in melancholic depression suggests that they remain valuable options in carefully selected patients [114,115].

The nuanced picture when comparing fluvoxamine and other antidepressants might be the result of the complex and heterogeneous nature of depressive disorders [116,117]. Fluvoxamine might show better effectiveness in some subgroups of patients. A recent prospective follow-up study showed that fluvoxamine significantly reduced interleukin-6 levels in major depressive disorder patients with high IL-6 baseline levels, indicating that the SSRI might be useful for patients with inflammatory depression, a subtype of depression featured by high incidence of treatment resistance [118,119]. Furthermore, in post-COVID-19 depression, SSRIs reduce depressive symptoms, and for these patients, fluvoxamine might be the drug of choice since studies have shown the benefit of fluvoxamine in preventing other clinical outcomes related to COVID-19 [35,54,120].

Moreover, genetic polymorphisms may influence individual response to antidepressants, including fluvoxamine. Genetic polymorphisms in genes expressing serotonergic receptors [89], serotonergic transporters [121,122], and neurotrophic factors [123] have been shown to impact fluvoxamine’s treatment response. Notably, fluvoxamine is primarily metabolized by the cytochrome P450 enzymes CYP2D6 and CYP1A2 [37]. Variations in the activity of these enzymes—particularly in individuals categorized as poor metabolizers or ultrarapid metabolizers—can lead to significant interindividual differences in plasma drug concentrations and therapeutic outcomes [124,125,126]. Poor metabolizers of CYP2D6 may exhibit increased plasma levels of fluvoxamine, potentially enhancing efficacy but also predisposing patients to concentration-dependent adverse effects, such as nausea, dizziness, sedation, or serotonin-mediated symptoms [124,125,126]. On the other hand, ultrarapid metabolizers may metabolize the drug too quickly, resulting in subtherapeutic exposure and insufficient clinical response [124]. Similarly, polymorphisms in CYP1A2, another major pathway in fluvoxamine metabolism, may alter drug clearance [127].

These pharmacogenomic insights suggest that CYP genotyping could support more individualized treatment decisions, for example, in CYP2D6 poor metabolizers, initiating treatment at lower doses or opting for antidepressants with more predictable pharmacokinetics may reduce the risk of adverse events. Conversely, ultrarapid metabolizers may require higher doses or different agents altogether to achieve therapeutic plasma concentrations [125,126].

This overview highlights fluvoxamine’s consistent efficacy across multiple comparisons, reinforcing its role as a viable option in the armamentarium of antidepressants. However, the heterogeneity of depression and the modest effect sizes across antidepressant trials suggest that no single medication is universally superior. Additionally, the emerging role of fluvoxamine’s sigma-1 receptor agonism warrants further exploration, particularly in addressing comorbid conditions like anxiety and post-traumatic stress disorder, where preliminary evidence suggests potential benefits [128,129]. Studies suggest that sigma-1 plays a pivotal role in the pathophysiology of depression and might be particularly involved in the cognitive symptoms of depression [130], suggesting that fluvoxamine may be an option for improving cognitive outcomes in depression [20].

Some limitations of our work should be acknowledged. First, although we included 14 systematic reviews, there was significant overlap in the RCTs analyzed across these reviews for most comparisons. Despite this, we identified head-to-head comparisons where results varied between reviews, demonstrating that including multiple systematic reviews helped capture some heterogeneity in the analyzed comparisons. Additionally, we attempted to include recent RCTs evaluating fluvoxamine’s efficacy in depression that were not part of previous systematic reviews, but none met our eligibility criteria. The high overlap between RCTs and variability in methods for estimating effect sizes precluded the execution of an additional meta-analysis on the extracted data. Second, while some systematic reviews conducted subgroup analyses (e.g., by gender or disease severity), this was not common across most reviews, limiting our ability to incorporate subgroup analyses into our work. Third, over half of the included systematic reviews did not achieve a high-quality rating on AMSTAR-2. However, we found that methodological quality scores were strongly correlated with publication year, indicating that more recent reviews generally adhere to higher methodological standards.

Despite these limitations, this comprehensive review concludes that fluvoxamine demonstrates strong efficacy in treating depression, comparable to other antidepressants and superior to placebo. Its distinct pharmacodynamic profile and consistent performance across studies highlight its clinical value. However, considering the heterogeneity of depression and the relatively small differences among antidepressants, individualized treatment strategies remain crucial. Further research is needed to clarify fluvoxamine’s role in emerging therapeutic contexts and to enhance its application in personalized psychiatry.

## 4. Methods

The protocol for this overview of systematic reviews was registered in the International Prospective Register of Systematic Reviews (PROSPERO) under registration number CRD42024557845 [131]. The study was performed following the Cochrane guidelines for Overviews of Reviews [132] and adhered to the Preferred Reporting Items for Systematic Reviews and Meta-Analyses (PRISMA) 2020 guidelines (for PRISMA checklist, see Table S1) [58].

### 4.1. Search and Selection

The following electronic databases were systematically searched to identify relevant studies: MEDLINE (PubMed) and the Cochrane Central Register of Controlled Trials. No limitations regarding language or year of publication were applied. The search was conducted using the following keywords: fluvoxamine AND (“mood disorder” OR depress* OR MDD OR dysthymia) AND (“systematic review” OR “meta-analysis”). Systematic reviews and meta-analyses of randomized controlled trials (open-label, single-blind, or double-blind) investigating the efficacy of fluvoxamine for treating depressive disorders were eligible. Participants were adults diagnosed with depressive disorders according to criteria established by the Diagnostic and Statistical Manual of Mental Disorders (DSM) or the International Classification of Diseases (ICD), who received fluvoxamine, placebo, or alternative medications. Diagnostic criteria from DSM-III to DSM-5, as well as ICD-10, were considered depending on the year each study was published.

Studies with the following characteristics were excluded: (1) narrative or non-systematic reviews; (2) reviews without confirmed standardized diagnoses using DSM or ICD criteria; (3) reviews restricted solely to populations outside the defined eligibility criteria (e.g., children, adolescents, elderly, pregnant women, individuals with perinatal anxiety or postpartum depression, COVID-19 patients, or COVID-19-associated syndromes); (4) reviews involving populations diagnosed with other psychiatric conditions or comorbidities; (5) reviews exclusively addressing fluvoxamine’s or other antidepressants’ tolerability or adverse effects; (6) reviews focusing solely on dose-response analyses; (7) reviews comparing fluvoxamine with different classes of medications or substances (e.g., benzodiazepines); (8) reviews comparing fluvoxamine exclusively to psychotherapeutic treatments; (9) reviews related exclusively to clinical practice guidelines; (10) reviews that did not individually assess fluvoxamine’s efficacy in terms of response or remission outcomes; (11) reviews without direct pairwise comparative efficacy data for fluvoxamine; (12) reviews involving preclinical research; (13) reviews solely focused on economic analyses of medications; and (14) systematic review protocols.

The study selection process was carried out in two phases: a screening phase (title and abstract review) and an eligibility phase (full-text review). Two investigators (Jair de Jesus Mari (JJM) and Thiago Wendt Viola (TWV)) independently conducted these phases, and any discrepancies were addressed and resolved through consensus with a third researcher (NRS). Agreement on study eligibility between the two investigators was assessed by calculating the Kappa coefficient, which indicated substantial concordance (κ = 0.709).

Additionally, a supplementary search was performed to identify individual randomized clinical trials (RCTs) not included in the eligible systematic reviews. This supplementary search used a cutoff date based on the latest inclusion date of the most recently published systematic review. MEDLINE was subsequently searched again, applying the original search strategy with systematic review-related terms removed and incorporating a clinical-trial-specific filter.

### 4.2. Data Extraction

After study selection, data were extracted independently by four analysts (Franciele Franco Scarante (FFS), JJM, Michel Haddad (MH), and Luiz Henrique Junqueira Dieckmann (LHJD)) and organized into a table summarizing the information from head-to-head comparisons involving fluvoxamine, placebo, and other drugs. The table contained the name of the first author, year of publication, identification of RCTs from each included review, number of participants analyzed, key findings, review type (pairwise meta-analysis, network meta-analysis [NMA], or umbrella review), and meta-analytic estimates, such as effect sizes and their respective dispersion measures. The extracted effect sizes included odds ratio (OR), relative risk (RR), mean difference (MD), rate difference (RD), relative benefit ratio (RBR), relative effect size (RES), standardized mean difference (SMD), and Hedge’s g (a measure quantifying standardized differences between group means [133]) accompanied by dispersion measures such as 95% confidence intervals (95% CI) or 95% credible intervals (95% CrI). For NMA data, only direct pairwise comparisons were extracted. Additionally, data from individual RCTs within each review were recorded, together with a qualitative summary of the primary results from each comparison (e.g., fluvoxamine vs. placebo, fluvoxamine vs. imipramine), clearly indicating whether each drug demonstrated superiority, equivalence, or inferiority.

To calculate the corrected covered area (CCA), which assesses the degree of overlap of primary studies included across the systematic reviews, we initially identified all primary studies appearing in the reviews and organized these into a matrix to record their occurrence. The CCA was subsequently calculated by evaluating study overlap between reviews, adjusting for both the total number of studies and reviews to avoid overestimating overlap due to review size. A given review was counted only once for each head-to-head comparison, even if it provided separate data on response and remission outcomes. Overlap results were expressed as percentages, with 6%–10% indicating “moderate overlap,” 11%–15% classified as “high overlap”, and greater than 15% as “very high overlap”.

### 4.3. Methodological Quality Assessment

The quality of the included reviews was evaluated using the “A Measurement Tool to Assess Systematic Reviews, version 2” (AMSTAR-2), specifically designed for rapid assessments of the methodological quality of systematic reviews of RCTs [134] (Table S2). Each review was assessed using all 16 items of the tool (Table S3). A Pearson’s correlation analysis was performed to assess the relationship between AMSTAR-2 scores and publication years of the reviews, with a significance threshold set at *p* < 0.05 (Figure S1). Reviews fulfilling all AMSTAR-2 criteria were classified as high quality. Reviews missing up to two criteria, typically those not reporting protocol registration or funding sources, were still considered high quality, acknowledging that these reporting practices have become increasingly standard over recent years. Reviews missing more criteria but still achieving a score higher than 10 were classified as moderate quality, while reviews with scores below 10 were categorized as low quality due to numerous methodological shortcomings. AMSTAR-2 assessments were initially conducted by one investigator (TWV), and the findings were subsequently discussed and confirmed with three other investigators (JJM, LHJD, and NRS).

## 5. Conclusions

This overview of systematic reviews and meta-analyses provides a comprehensive synthesis of the efficacy of fluvoxamine in the treatment of depressive disorders. The evidence consistently shows that fluvoxamine is significantly more effective than placebo in achieving clinical response and remission. When compared to other antidepressants—including tricyclic antidepressants, other SSRIs, SNRIs, and atypical agents—fluvoxamine demonstrated comparable efficacy, with some differences favoring specific agents such as venlafaxine and mirtazapine in isolated comparisons.

Given its unique pharmacological properties, including its action as a sigma-1 receptor agonist, fluvoxamine may offer therapeutic advantages in specific patient populations, especially where comorbid conditions such as anxiety, obsessive-compulsive disorder, or post-traumatic stress disorder are present. These findings support the continued clinical use of fluvoxamine and highlight the need for future studies focused on its role within personalized treatment strategies and multimorbidity contexts.

## Figures and Tables

**Table 1:** Characteristics of high-quality reviews and head-to-head comparisons on the efficacy of fluvoxamine assessed by treatment response. Abbreviations: CI, confidence interval; db, double-blind; NMA, network meta-analysis; MA, meta-analysis; ol, open label; OR, odds ratio; RCT, randomized clinical trial; RR, relative risk; SSRIs, selective serotonin reuptake inhibitors; SNRIs, serotonin and norepinephrine reuptake inhibitors; TCA, tricyclic antidepressants.

Review	Type	Number of Included RCTs (Number of Patients)	Time of Follow-Up (Weeks)	Blinding RCTs	Summary Estimates (ES, 95% CI)	Main Findings
Fluvoxamine vs. Placebo						
Cipriani et al., 2018 [49]	NMA	14 (1799)	4–6	db	OR = 1.69 (1.41, 2.02)	Fluvoxamine significantly more effective than placebo
Fluvoxamine vs. TCA Unspecified TCA						
Omori et al., 2010 [40]	MA	16 (935)	6–10	db	OR = 0.97 (0.73, 1.29)	Non-significant difference
Omori et al., 2009 [39]	MA	16 (872)	6–10	db	RR = 0.99 (0.86, 1.14)	Non-significant difference
Imipramine						
Omori et al., 2010 [40]	MA	7 (422)	6	db	OR = 0.97 (0.59, 1,58)	Non-significant difference
Omori et al., 2009 [39]	MA	6 (282)	6	db	RR = 0.95 (0.67, 1.36)	Non-significant difference
Clomipramine						
Omori et al., 2010 [40]	MA	2 (159)	6–8	db	OR = 0.84 (0.38, 1.85)	Non-significant difference
Omori et al., 2009 [39]	MA	1 (86)	8	db	RR = 0.99 (0.68, 1.44)	Non-significant difference
Cipriani et al., 2018 [49]	NMA	2 (83)	4–6	db	OR = 1.01 (0.76, 1.32)	Non-significant difference
Amitriptyline						
Omori et al., 2010 [40]	MA	4 (185)	6–7	ol and db	OR = 0.79 (0.35, 1.75)	Non-significant difference
Omori et al., 2009 [39]	MA	4 (185)	6–7	ol and db	RR = 0.91 (0.61, 1.38)	Non-significant difference
Cipriani et al., 2018 [49]	NMA	3 (337)	4–7	db	OR = 1.25 (0.99, 1.59)	Non-significant difference
Dothiepin						
Omori et al., 2009 [39]	MA	2 (125)	6	db	RR = 1.05 (0.65, 1.69)	Non-significant difference
Omori et al., 2010 [40]	MA	2 (125)	6	db	OR = 1.11 (0.55, 2.24)	Non-significant difference
Desipramine						
Omori et al., 2010 [40]	MA	1 (47)	10	db	OR = 4.22 (0.98, 18.13)	Non-significant difference
Omori et al., 2009 [39]	MA	1 (47)	10	db	RR = 1.44 (0.90, 2.31)	Non-significant difference
Nortriptyline						
Omori et al., 2010 [40]	MA	1 (74)	8	ol	OR = 0.91 (0.36, 2.28)	Non-significant difference
Omori et al., 2009 [39]	MA	1 (74)	8	ol	OR = 0.96 (0.57, 1.62)	Non-significant difference
Fluvoxamine vs. SSRIs Unspecified SSRIs						
Omori et al., 2010 [40]	MA	8 (967)	6–7	db	OR = 0.96 (0.74, 1.25)	Non-significant difference
Paroxetine						
Omori et al., 2010 [40]	MA	3 (281)	6–7	ol and db	OR = 0.83 (0.51, 1.34)	Non-significant difference
Omori et al., 2009 [39]	MA	3 (281)	6–7	ol and db	RR = 0.92 (0.70, 1.21)	Non-significant difference
Cipriani et al., 2009 [46]	MA	3 (260)	6–7	db	OR = 0.83 (0.51, 1.34)	Non-significant difference
Cipriani et al., 2018 [49]	NMA	2 (180)	6–7	db	OR= 0.84 (0.67, 1.04)	Non-significant difference
Sertraline						
Omori et al., 2010 [40]	MA	2 (185)	7	db	OR = 1.21 (0.53, 2.75)	Non-significant difference
Omori et al., 2009 [39]	MA	2 (185)	7	db	RR = 1.10 (0.71, 1.70)	Non-significant difference
Cipriani et al., 2009 [46]	NMA	2 (185)	7	db	OR = 1.21 (0.53, 2.75)	Sertraline significantly more effective than fluvoxamine
Cipriani et al., 2018 [49]	NMA	2 (185)	7	db	OR = 0.89 (0.70, 1.13)	Non-significant difference
Fluoxetine						
Omori et al., 2009 [39]	MA	2 (284)	6–7	db	RR = 1.00 (0.78, 1.28)	Non-significant difference
Omori et al., 2010 [40]	MA	2 (284)	6–7	db	OR = 1.00 (0.62, 1.61)	Non-significant difference
Cipriani et al., 2009 [46]	MA	2 (284)	6–7	db	OR = 1.03 (0.64, 1.66)	Non-significant difference
Cipriani et al., 2018 [49]	NMA	2 (284)	6–7	db	OR= 1.00 (0.80, 1.25)	Non-significant difference
Citalopram						
Omori et al., 2010 [40]	MA	1 (217)	6	db	OR = 0.90 (0.50, 1.62)	Non-significant difference
Omori et al., 2009 [39]	MA	1 (217)	6	db	RR = 0.93 (0.54, 1.60)	Non-significant difference
Cipriani et al., 2009 [46]	MA	1 (217)	6	db	OR = 0.90 (0.50, 1.62)	Non-significant difference
Cipriani et al., 2018 [49]	NMA	1 (217)	6	db	OR = 1.06 (0.82, 1.39)	Non-significant difference
Fluvoxamine vs. SNRIs Unspecified SNRIs						
Omori et al., 2010 [40]	MA	3 (258)	6	db	OR = 0.48 (0.27, 0.85)	SNRIs significantly more effective than fluvoxamine
Omori et al., 2009 [39]	MA	2 (224)	6	db	RR = 0.76 (0.56, 1.04)	Non-significant difference
Milnacipran						
Omori et al., 2010 [40]	MA	1 (113)	6	db	OR = 0.57 (0.26, 1.23)	Non-significant difference
Omori et al., 2009 [39]	MA	1 (113)	6	db	RR = 0.81 (0.56, 1.18)	Non-significant difference
Nakagawa et al., 2009 [47]	MA	1 (113)	6	db	OR = 1.76 (0.81, 3.83)	Non-significant difference
Cipriani et al., 2009 [46]	MA	1 (113)	6	db	OR = 0.57 (0.26, 1.23)	Non-significant difference
Cipriani et al., 2018 [49]	NMA	2 (239)	4–6	db	OR = 0.89 (0.67, 1.17)	Non-significant difference
Venlafaxine						
Cipriani et al., 2009 [46]	MA	1 (71)	6	db	OR = 0.42 (0.19, 0.96)	Venlafaxine significantly more effective than fluvoxamine
Omori et al., 2010 [40]	MA	2 (145)	6	db	OR = 0.40 (0.18, 0.92)	Fluvoxamine less effective than venlafaxine
Omori et al., 2009 [39]	MA	1 (111)	6	db	RR = 0.65 (0.37, 1.15)	Non-significant difference
Cipriani et al., 2018 [49]	NMA	2 (111)	6	db	OR = 0.84 (0.66, 1.07)	Non-significant difference
Fluvoxamine vs. Other Antidepressants Mirtazapine						
Omori et al., 2009 [39]	MA	1 (412)	6	db	RR = 0.95 (0.78, 1.16)	Non-significant difference
Omori et al., 2010 [40]	MA	1 (412)	6	db	OR = 0.72 (0.47, 1.11)	Non-significant difference
Cipriani et al., 2009 [46]	MA	1 (412)	6	db	OR = 0.88 (0.59, 1.31)	Mirtazapine significantly more effective than fluvoxamine
Cipriani et al., 2018 [49]	NMA	2 (412)	6	db	OR= 0.78 (0.60, 0.99)	Mirtazapine superior to fluvoxamine
Mianserin						
Omori et al., 2009 [39]	MA	2 (125)	6	db	RR = 1.09 (0.86, 1.40)	Non-significant difference
Omori et al. 2010 [40]	MA	2 (125)	6	db	OR = 1.25 (0.55, 2.87)	Non-significant difference

**Table 2:** Characteristics of high-quality reviews and head-to-head comparisons on the efficacy of fluvoxamine assessed by remission. Abbreviations: CI, confidence interval; db, double-blind; NMA, network meta-analysis; MA, meta-analysis; ol, open label; OR, odds ratio; RCT, randomized clinical trial; RR, relative risk; SSRIs, selective serotonin reuptake inhibitors; SNRIs, serotonin and norepinephrine reuptake inhibitors; TCA, tricyclic antidepressants.

Review	Type	Number of Included RCTs (Number of Patients)	Time of Follow-Up (Weeks)	Blinding RCTs	Summary Estimates (ES, 95% CI)	Main Findings
Fluvoxamine vs. Placebo						
Kishi et al., 2023 [52]	NMA	1 (204)	52	db	RR = 0.298 (0.114, 0.686) *	Fluvoxamine significantly more effective than placebo
Cipriani et al., 2018 [49]	NMA	14 (1799) **	4–6	db	OR = 0.58 (0.39, 0.86)	Fluvoxamine significantly more effective than placebo
Fluvoxamine vs. TCA Unspecified TCA						
Omori et al., 2010 [40]	MA	16 (965)	6–10	db	OR = 1.00 (0.69, 1.45)	Non-significant difference
Omori et al., 2009 [39]	MA	16 (872)	6–10	db	RR = 0.98 (0.71, 1.35)	Non-significant difference
Imipramine						
Omori et al., 2010 [40]	MA	6 (375)	6	db	OR = 1.07 (0.59, 1.94)	Non-significant difference
Omori et al., 2009 [39]	MA	6 (282)	6	db	RR = 1.03 (0.53, 2.00)	Non-significant difference
Clomipramine						
Omori et al., 2010 [40]	MA	2 (159)	6–8	db	OR = 0.64 (0.28, 1.49)	Non-significant difference
Omori et al., 2009 [39]	MA	1 (86)	8	db	RR = 0.72 (0.20, 2.56)	Non-significant difference
Cipriani et al., 2018 [49]	NMA	2 (83) **	4–6	db	OR = 1.57 (0.56, 4.57)	Non-significant difference
Amitriptyline						
Omori et al., 2010 [40]	MA	4 (185)	6–7	ol and db	OR = 0.61 (0.28, 1.31)	Non-significant difference
Omori et al., 2009 [39]	MA	4 (185)	6–7	ol and db	RR = 0.74 (0.42, 1.30)	Non-significant difference
Cipriani et al., 2018 [49]	NMA	3 (337) **	4–7	db	OR = 1.07 (0.61, 1.88)	Non-significant difference
Dothiepin						
Omori et al., 2009 [39]	MA	2 (125)	6	db	RR = 1.05 (0.48, 2.25)	Non-significant difference
Omori et al., 2010 [40]	MA	2 (125)	6	db	OR = 1.06 (0.48, 2.35)	Non-significant difference
Desipramine						
Omori et al., 2010 [40]	MA	1 (47)	10	db	OR = 4.5 (1.31, 15.42)	Fluvoxamine significantly more effective than desipramine
Omori et al., 2009 [39]	MA	1 (47)	10	db	RR = 2.27 (0.90, 5.73)	Non-significant difference
Nortriptyline						
Omori et al., 2010 [40]	MA	1 (74)	8	ol	OR = 1.78 (0.67, 4.77)	Non-significant difference
Omori et al., 2009 [39]	MA	1 (74)	8	ol	OR = 1.48 (0.61, 3.57)	Non-significant difference
Fluvoxamine vs. SSRIs Unspecified SSRIs						
Omori et al., 2010 [40]	MA	8 (967)	6–7	db	OR = 0.98 (0.71, 1.37)	Non-significant difference
Paroxetine						
Omori et al., 2010 [40]	MA	3 (281)	6–7	ol and db	OR = 0.77 (0.45, 1.33)	Non-significant difference
Omori et al., 2009 [39]	MA	3 (281)	6–7	ol and db	RR = 0.83 (0.52, 1.31)	Non-significant difference
Cipriani et al., 2018 [49]	NMA	2 (180) **	6–7	db	OR= 1.13 (0.50, 2.46)	Non-significant difference
Sertraline						
Omori et al., 2010 [40]	MA	2 (185)	7	db	OR = 1.31 (0.48, 3.57)	Non-significant difference
Omori et al., 2009 [39]	MA	2 (185)	7	db	RR = 1.10 (0.63, 2.15)	Non-significant difference
Ramsberg et al., 2012 [48]	NMA	1 (88)	7	db	OR = 1.41 (0.92, 2.10) ***	Non-significant difference
Cipriani et al., 2018 [49]	NMA	2 (185) **	7	db	OR = 0.68 (0.34, 1.36)	Non-significant difference
Fluoxetine						
Omori et al., 2009 [39]	MA	2 (284)	6–7	db	RR = 1.15 (0.72, 1.82)	Non-significant difference
Omori et al., 2010 [40]	MA	2 (284)	6–7	db	OR = 1.24 (0.74, 2.06)	Non-significant difference
Cipriani et al., 2018 [49]	NMA	2 (284) **	6–7	db	OR = 0.85 (0.47, 1.51)	Non-significant difference
Citalopram						
Omori et al., 2010 [40]	MA	1 (217)	6	db	OR = 0.56 (0.23, 1.34)	Non-significant difference
Omori et al., 2009 [39]	MA	1 (217)	6	db	RR = 0.59 (0.21, 1.66)	Non-significant difference
Ramsberg et al., 2012 [48]	NMA	1 (217)	6	db	OR = 0.80 (0.51, 1.19) ***	Non-significant difference
Cipriani et al., 2018 [49]	NMA	1 (217) **	6	db	OR = 1.84 (0.72, 5.00)	Non-significant difference
Fluvoxamine vs. SNRIs Unspecified SNRIs						
Omori et al., 2010 [40]	MA	3 (258)	6	db	OR = 0.61 (0.34, 1.08)	Non-significant difference
Omori et al., 2009 [39]	MA	2 (224)	6	db	RR = 0.73 (0.45, 1.20)	Non-significant difference
Milnacipram						
Omori et al., 2010 [40]	MA	1 (113)	6	db	OR = 0.68 (0.3, 1.51)	Non-significant difference
Omori et al., 2009 [39]	MA	1 (113)	6	db	RR = 0.76 (0.37, 1.59)	Non-significant difference
Nakagawa et al., 2009 [47]	MA	1 (113)	6	db	OR = 1.48 (0.66, 3.3)	Non-significant difference
Lopez-Ibor et al., 1996 [43]	MA	1 (113)	6	db	Milnacipram = 47% Fluvoxamine = 36% *p* = 0.20	Non-significant difference
Venlafaxine						
Omori et al., 2010 [40]	MA	2 (145)	6	db	OR = 0.54 (0.23, 1.24)	Non-significant difference
Omori et al., 2009 [39]	MA	1 (111)	6	db	RR = 0.70 (0.36, 1.37)	Non-significant difference
Cipriani et al., 2018 [49]	NMA	2 (111) **	6.	db	OR = 1.93 (0.76, 5.01)	Non-significant difference
Fluvoxamine vs. Other Antidepressants Mirtazapine						
Omori et al., 2009 [39]	MA	1 (412)	6	db	RR = 1.10 (0.83, 1.45)	Non-significant difference
Omori et al., 2010 [40]	MA	1 (412)	6	db	OR = 1.19 (0.81, 1.76)	Non-significant difference
Cipriani et al., 2018 [49]	NMA	2 (412) **	6	db	OR = 0.84 (0.52, 1.36)	Non-significant difference
Mianserin						
Omori et al., 2009 [39]	MA	2 (125)	6	db	RR = 1.16 (0.93, 1.44)	Non-significant difference
Omori et al. 2010 [40]	MA	2 (125)	6	db	OR = 2.02 (0.55, 7.39)	Non-significant difference

* The outcome measured on Kishi et al., 2023 was six-month relapse rate. ** Specific information about the RCTs included in the remission analysis by Cipriani et al., (2018) was not reported. *** The outcome measured on Ramsberg et al., 2012 was probability of remission.

## Data Availability

The original contributions presented in this study are included in the article/Supplementary Materials. Further inquiries can be directed to the corresponding author.

## Supplementary Materials

The following supporting information can be downloaded at: https://www.mdpi.com/article/10.3390/ph18050711/s1, Table S1: PRISMA Checklist; Table S2: Assessment of methodological quality of the included systematic reviews using the tool “A Measurement Tool to Assess Systematic Reviews, version 2” (AMSTAR-2); Table S3. Items and questions of the AMSTAR-2 used on the assessment of methodological quality of the included systematic reviews; Table S4: Comprehensive summary of the overview of reviews, encompassing all findings from the included reviews, independently of the AMSTAR classification; Figure S1. Pearson’s Correlation between AMSTAR-2 scores and publication year of the included reviews. References [135,136,137,138,139,140,141,142,143,144,145,146,147,148,149,150,151] are cited in Supplementary Materials.

## References

[1] GBD 2019 Mental Disorders Collaborators (2022). Global, regional, and national burden of 12 mental disorders in 204 countries and territories, 1990–2019: A systematic analysis for the Global Burden of Disease Study 2019. Lancet Psychiatry.

[2] Thornicroft G., Chatterji S., Evans-Lacko S., Gruber M., Sampson N., Aguilar-Gaxiola S., Al-Hamzawi A., Alonso J., Andrade L., Borges G. (2017). Undertreatment of people with major depressive disorder in 21 countries. Br. J. Psychiatry.

[3] Malhi G.S., Mann J.J. (2018). Depression. Lancet.

[4] Shorey S., Ng E.D., Wong C.H.J. (2022). Global prevalence of depression and elevated depressive symptoms among adolescents: A systematic review and meta-analysis. Br. J. Clin. Psychol..

[5] Salk R.H., Hyde J.S., Abramson L.Y. (2017). Gender differences in depression in representative national samples: Meta-analyses of diagnoses and symptoms. Psychol. Bull..

[6] Gobbi G., Atkin T., Zytynski T., Wang S., Askari S., Boruff J., Ware M., Marmorstein N., Cipriani A., Dendukuri N. (2019). Association of Cannabis Use in Adolescence and Risk of Depression, Anxiety, and Suicidality in Young Adulthood: A Systematic Review and Meta-analysis. JAMA Psychiatry.

[7] Zenebe Y., Akele B., W/Selassie M., Necho M. (2021). Prevalence and determinants of depression among old age: A systematic review and meta-analysis. Ann. Gen. Psychiatry.

[8] Szymkowicz S.M., Gerlach A.R., Homiack D., Taylor W.D. (2023). Biological factors influencing depression in later life: Role of aging processes and treatment implications. Transl. Psychiatry.

[9] Beijers L., Wardenaar K.J., van Loo H.M., Schoevers R.A. (2019). Data-driven biological subtypes of depression: Systematic review of biological approaches to depression subtyping. Mol. Psychiatry.

[10] Musil R., Seemüller F., Meyer S., Spellmann I., Adli M., Bauer M., Kronmüller K.T., Brieger P., Laux G., Bender W. (2018). Subtypes of depression and their overlap in a naturalistic inpatient sample of major depressive disorder. Int. J. Methods Psychiatr. Res..

[11] Lamers F., Vogelzangs N., Merikangas K.R., de Jonge P., Beekman A.T., Penninx B.W. (2013). Evidence for a differential role of HPA-axis function, inflammation and metabolic syndrome in melancholic versus atypical depression. Mol. Psychiatry.

[12] Spiegler G., Su Y., Li M., Wolfson C., Meng X., Schmitz N. (2024). Characterization of depression subtypes and their relationships to stressor profiles among middle-aged and older adults: An analysis of the canadian longitudinal study on aging (CLSA). J. Psychiatr. Res..

[13] Tozzi L., Zhang X., Pines A., Olmsted A.M., Zhai E.S., Anene E.T., Chesnut M., Holt-Gosselin B., Chang S., Stetz P.C. (2024). Personalized brain circuit scores identify clinically distinct biotypes in depression and anxiety. Nat. Med..

[14] Hillhouse T.M., Porter J.H. (2015). A brief history of the development of antidepressant drugs: From monoamines to glutamate. Exp. Clin. Psychopharmacol..

[15] Chang J.P.-C., Zamparelli A., Nettis M.A., Pariante C.M., Della Sala S. (2022). Antidepressant Drugs: Mechanisms of Action and Side Effects. Encyclopedia of Behavioral Neuroscience.

[16] Saletu B., Schjerve M., Grünberger J., Schanda H., Arnold O.H. (1977). Fluvoxamine-a new serotonin re-uptake inhibitor: First clinical and psychometric experiences in depressed patients. J. Neural Transm..

[17] Claassen V. (1983). Review of the animal pharmacology and pharmacokinetics of fluvoxamine. Br. J. Clin. Pharmacol..

[18] Hrdina P.D. (1991). Pharmacology of serotonin uptake inhibitors: Focus on fluvoxamine. J. Psychiatry Neurosci..

[19] Ago Y., Hasebe S., Hiramatsu N., Hashimoto H., Takuma K., Matsuda T. (2017). Psychopharmacology of combined activation of the serotonin. Eur. J. Pharmacol..

[20] Hindmarch I., Hashimoto K. (2010). Cognition and depression: The effects of fluvoxamine, a sigma-1 receptor agonist, reconsidered. Hum. Psychopharmacol..

[21] Ishikawa M., Ishiwata K., Ishii K., Kimura Y., Sakata M., Naganawa M., Oda K., Miyatake R., Fujisaki M., Shimizu E. (2007). High occupancy of sigma-1 receptors in the human brain after single oral administration of fluvoxamine: A positron emission tomography study using [11C]SA4503. Biol. Psychiatry.

[22] Ren P., Wang J., Li N., Li G., Ma H., Zhao Y., Li Y. (2022). Sigma-1 Receptors in Depression: Mechanism and Therapeutic Development. Front. Pharmacol..

[23] Wang Y.M., Xia C.Y., Jia H.M., He J., Lian W.W., Yan Y., Wang W.P., Zhang W.K., Xu J.K. (2022). Sigma-1 receptor: A potential target for the development of antidepressants. Neurochem. Int..

[24] Dobrodeeva V., Abdyrahmanova A., Astafeva D., Smirnova D., Cumming P., De Sousa A., Davydkin I., Yashikhina A., Shnayder N., Nasyrova R. (2022). Pharmacogenetic Aspects of COVID-19 Management and Post-COVID-19 Depression Treatment with Fluvoxamine. Psychiatr. Danub..

[25] Lenze E.J., Reiersen A.M., Santosh P.J. (2022). Repurposing fluvoxamine, and other psychiatric medications, for COVID-19 and other conditions. World Psychiatry.

[26] Hashimoto Y., Suzuki T., Hashimoto K. (2022). Mechanisms of action of fluvoxamine for COVID-19: A historical review. Mol. Psychiatry.

[27] McIntyre R.S., Alsuwaidan M., Baune B.T., Berk M., Demyttenaere K., Goldberg J.F., Gorwood P., Ho R., Kasper S., Kennedy S.H. (2023). Treatment-resistant depression: Definition, prevalence, detection, management, and investigational interventions. World Psychiatry.

[28] Rush A.J., Trivedi M.H., Wisniewski S.R., Nierenberg A.A., Stewart J.W., Warden D., Niederehe G., Thase M.E., Lavori P.W., Lebowitz B.D. (2006). Acute and longer-term outcomes in depressed outpatients requiring one or several treatment steps: A STAR*D report. Am. J. Psychiatry.

[29] McAllister-Williams R.H., Arango C., Blier P., Demyttenaere K., Falkai P., Gorwood P., Hopwood M., Javed A., Kasper S., Malhi G.S. (2020). The identification, assessment and management of difficult-to-treat depression: An international consensus statement. J. Affect. Disord..

[30] Gammoh O.S., Bashatwah R. (2023). Potential strategies to optimize the efficacy of antidepressants: Beyond the monoamine theory. Electron. J. Gen. Med..

[31] Haddad M., Dieckmann L.H.J., Viola T.W., de Araújo M.R., da Silva N.R., Mari J.J. (2025). The Efficacy of Fluvoxamine in Anxiety Disorders and Obsessive-Compulsive Disorder: An Overview of Systematic Reviews and Meta-Analyses. Pharmaceuticals.

[32] European Medicines Agency Floxyfral Registration. https://www.ema.europa.eu/en/documents/referral/summary-information-referral-opinion-following-arbitration-pursuant-article-30-council-directive-200183ec-floxyfral-and-associated-names-international-non-proprietary-name-inn-fluvoxamine-background_en.pdf.

[33] FDA Label—Fluvoxamine (Luvox^®^) Prescribing Information. https://www.accessdata.fda.gov/drugsatfda_docs/label/2008/022235lbl.pdf.

[34] Williams T., McCaul M., Schwarzer G., Cipriani A., Stein D.J., Ipser J. (2020). Pharmacological treatments for social anxiety disorder in adults: A systematic review and network meta-analysis. Acta Neuropsychiatr..

[35] Zhou Q., Zhao G., Pan Y., Zhang Y., Ni Y. (2024). The efficacy and safety of fluvoxamine in patients with COVID-19: A systematic review and meta-analysis from randomized controlled trials. PLoS ONE.

[36] van Harten J. (1995). Overview of the pharmacokinetics of fluvoxamine. Clin. Pharmacokinet..

[37] Perucca E., Gatti G., Spina E. (1994). Clinical pharmacokinetics of fluvoxamine. Clin. Pharmacokinet..

[38] Burhanuddin K., Badhan R. (2022). Optimising Fluvoxamine Maternal/Fetal Exposure during Gestation: A Pharmacokinetic Virtual Clinical Trials Study. Metabolites.

[39] Omori I.M., Watanabe N., Nakagawa A., Akechi T., Cipriani A., Barbui C., McGuire H., Churchill R., Furukawa T.A., Meta-Analysis of New Generation Antidepressants (MANGA) Study Group (2009). Efficacy, tolerability and side-effect profile of fluvoxamine for major depression: Meta-analysis. J. Psychopharmacol..

[40] Omori I.M., Watanabe N., Nakagawa A., Cipriani A., Barbui C., McGuire H., Churchill R., Furukawa T.A. (2010). Fluvoxamine versus other anti-depressive agents for depression. Cochrane Database Syst. Rev..

[41] Anderson I.M., Tomenson B.M. (1994). The efficacy of selective serotonin re-uptake inhibitors in depression: A meta-analysis of studies against tricyclic antidepressants. J. Psychopharmacol..

[42] Möller H.J., Fuger J., Kasper S. (1994). Efficacy of new generation antidepressants: Meta-analysis of imipramine-controlled studies. Pharmacopsychiatry.

[43] Lopez-Ibor J., Guelfi J.D., Pletan Y., Tournoux A., Prost J.F. (1996). Milnacipran and selective serotonin reuptake inhibitors in major depression. Int. Clin. Psychopharmacol..

[44] Anderson I.M. (2000). Selective serotonin reuptake inhibitors versus tricyclic antidepressants: A meta-analysis of efficacy and tolerability. J. Affect. Disord..

[45] Nemeroff C.B., Entsuah R., Benattia I., Demitrack M., Sloan D.M., Thase M.E. (2008). Comprehensive analysis of remission (COMPARE) with venlafaxine versus SSRIs. Biol. Psychiatry.

[46] Cipriani A., Furukawa T.A., Salanti G., Geddes J.R., Higgins J.P., Churchill R., Watanabe N., Nakagawa A., Omori I.M., McGuire H. (2009). Comparative efficacy and acceptability of 12 new-generation antidepressants: A multiple-treatments meta-analysis. Lancet.

[47] Nakagawa A., Watanabe N., Omori I.M., Barbui C., Cipriani A., McGuire H., Churchill R., Furukawa T.A. (2009). Milnacipran versus other antidepressive agents for depression. Cochrane Database Syst. Rev..

[48] Ramsberg J., Asseburg C., Henriksson M. (2012). Effectiveness and cost-effectiveness of antidepressants in primary care: A multiple treatment comparison meta-analysis and cost-effectiveness model. PLoS ONE.

[49] Cipriani A., Furukawa T.A., Salanti G., Chaimani A., Atkinson L.Z., Ogawa Y., Leucht S., Ruhe H.G., Turner E.H., Higgins J.P.T. (2018). Comparative efficacy and acceptability of 21 antidepressant drugs for the acute treatment of adults with major depressive disorder: A systematic review and network meta-analysis. Lancet.

[50] Yuan Z., Chen Z., Xue M., Zhang J., Leng L. (2020). Application of antidepressants in depression: A systematic review and meta-analysis. J. Clin. Neurosci..

[51] Suchting R., Tirumalajaru V., Gareeb R., Bockmann T., de Dios C., Aickareth J., Pinjari O., Soares J.C., Cowen P.J., Selvaraj S. (2021). Revisiting monoamine oxidase inhibitors for the treatment of depressive disorders: A systematic review and network meta-analysis. J. Affect. Disord..

[52] Kishi T., Ikuta T., Sakuma K., Okuya M., Hatano M., Matsuda Y., Iwata N. (2023). Antidepressants for the treatment of adults with major depressive disorder in the maintenance phase: A systematic review and network meta-analysis. Mol. Psychiatry.

[53] Nematizadeh M., Ghorbanzadeh H., Moghaddam H.S., Shalbafan M., Boroon M., Keshavarz-Akhlaghi A.A., Akhondzadeh S. (2023). L-theanine combination therapy with fluvoxamine in moderate-to-severe obsessive-compulsive disorder: A placebo-controlled, double-blind, randomized trial. Psychiatry Clin. Neurosci..

[54] Farahani R.H., Ajam A., Naeini A.R. (2023). Effect of fluvoxamine on preventing neuropsychiatric symptoms of post COVID syndrome in mild to moderate patients, a randomized placebo-controlled double-blind clinical trial. BMC Infect. Dis..

[55] She D.P., He Y., Li M.Q., Su L., Ren D., Huang X.H., Zhang Y.H., Hu H.T., Deng D.C., Wu J.L. (2023). Pharmacokinetics and bioequivalence studies of fluvoxamine maleate tablets in healthy Chinese subjects. Biomed. Chromatogr..

[56] Tsujii T., Sakurai H., Takeuchi H., Suzuki T., Mimura M., Uchida H. (2022). Predictors of response to pharmacotherapy in children and adolescents with psychiatric disorders: A combined post hoc analysis of four clinical trial data. Neuropsychopharmacol. Rep..

[57] Brar J., Sidana A., Chauhan N., Bajaj M.K. (2022). A randomized, open-label pilot trial of selective serotonin reuptake inhibitors on neuropsychological functions in patients with obsessive compulsive disorder. J. Psychiatr. Res..

[58] Page M.J., McKenzie J.E., Bossuyt P.M., Boutron I., Hoffmann T.C., Mulrow C.D., Shamseer L., Tetzlaff J.M., Akl E.A., Brennan S.E. (2021). The PRISMA 2020 statement: An updated guideline for reporting systematic reviews. BMJ.

[59] Amin M.M., Ananth J.V., Coleman B.S., Darcourt G., Farkas T., Goldstein B., Lapierre Y.D., Paykel E., Wakelin J.S. (1984). Fluvoxamine: Antidepressant effects confirmed in a placebo-controlled international study. Clin. Neuropharmacol..

[60] Brown W.A., Arato M., Shrivastava R. (1986). Pituitary-adrenocortical hyperfunction and intolerance to fluvoxamine, a selective serotonin uptake inhibitor. Am. J. Psychiatry.

[61] Cassano G.B., Conti L., Massimetti G., Mengali F., Waekelin J.S., Levine J. (1986). Use of a standardized documentation system (BLIPS/BDP) in the conduct of a multicenter international trial comparing fluvoxamine, imipramine, and placebo. Psychopharmacol. Bull..

[62] Claghorn J.L., Earl C.Q., Walczak D.D., Stoner K.A., Wong L.F., Kanter D., Houser V.P. (1996). Fluvoxamine maleate in the treatment of depression: A single-center, double-blind, placebo-controlled comparison with imipramine in outpatients. J. Clin. Psychopharmacol..

[63] Dominguez R.A., Goldstein B.J., Jacobson A.F., Steinbook R.M. (1985). A double-blind placebo-controlled study of fluvoxamine and imipramine in depression. J. Clin. Psychiatry.

[64] Fabre L., Birkhimer L.J., Zaborny B.A., Wong L.F., Kapik B.M. (1996). Fluvoxamine versus imipramine and placebo: A double-blind comparison in depressed patients. Int. Clin. Psychopharmacol..

[65] Feighner J.P., Boyer W.F., Meredith C.H., Hendrickson G.G. (1989). A placebo-controlled inpatient comparison of fluvoxamine maleate and imipramine in major depression. Int. Clin. Psychopharmacol..

[66] Itil T.M., Shrivastava R.K., Mukherjee S., Coleman B.S., Michael S.T. (1983). A double-blind placebo-controlled study of fluvoxamine and imipramine in out-patients with primary depression. Br. J. Clin. Pharmacol..

[67] Lapierre Y.D., Browne M., Horn E., Oyewumi L.K., Sarantidis D., Roberts N., Badoe K., Tessier P. (1987). Treatment of major affective disorder with fluvoxamine. J. Clin. Psychiatry.

[68] Lydiard R.B., Laird L.K., Morton W.A., Steele T.E., Kellner C., Laraia M.T., Ballenger J.C. (1989). Fluvoxamine, imipramine, and placebo in the treatment of depressed outpatients: Effects on depression. Psychopharmacol. Bull..

[69] March J.S., Kobak K.A., Jefferson J.W., Mazza J., Greist J.H. (1990). A double-blind, placebo-controlled trial of fluvoxamine versus imipramine in outpatients with major depression. J. Clin. Psychiatry.

[70] Norton K.R., Sireling L.I., Bhat A.V., Rao B., Paykel E.S. (1984). A double-blind comparison of fluvoxamine, imipramine and placebo in depressed patients. J. Affect. Disord..

[71] Roth D., Mattes J., Sheehan K.H., Sheehan D.V. (1990). A double-blind comparison of fluvoxamine, desipramine and placebo in outpatients with depression. Prog. Neuropsychopharmacol. Biol. Psychiatry.

[72] Walczak D.D., Apter J.T., Halikas J.A., Borison R.L., Carman J.S., Post G.L., Patrick R., Cohn J.B., Cunningham L.A., Rittberg B. (1996). The oral dose-effect relationship for fluvoxamine: A fixed-dose comparison against placebo in depressed outpatients. Ann. Clin. Psychiatry.

[73] Terra J.L., Montgomery S.A. (1998). Fluvoxamine prevents recurrence of depression: Results of a long-term, double-blind, placebo-controlled study. Int. Clin. Psychopharmacol..

[74] Guy W., Wilson W.H., Ban T.A., King D.L., Manov G., Fjetland O.K. (1984). A double-blind clinical trial of fluvoxamine and imipramine in patients with primary depression. Psychopharmacol. Bull..

[75] Miller H.L., Ekstrom R.D., Mason G.A., Lydiard R.B., Golden R.N. (2001). Noradrenergic function and clinical outcome in antidepressant pharmacotherapy. Neuropsychopharmacology.

[76] Zohar J., Keegstra H., Barrelet L. (2003). Fluvoxamine as effective as clomipramine against symptoms of severe depression: Results from a multicentre, double-blind study. Hum. Psychopharmacol..

[77] De Wilde J.E., Mertens C., Wakelin J.S. (1983). Clinical trials of fluvoxamine vs chlorimipramine with single and three times daily dosing. Br. J. Clin. Pharmacol..

[78] Ottevanger E.A. (1995). Fluvoxamine and clomipramine in depressed hospitalised patients: Results from a randomised, double-blind study. Encephale.

[79] Barge-Schaapveld D.Q., Nicolson N.A., van der Hoop R.G., De Vries M.W. (1995). Changes in daily life experience associated with clinical improvement in depression. J. Affect. Disord..

[80] Harris B., Szulecka T.K., Anstee J.A. (1991). Fluvoxamine versus amitriptyline in depressed hospital out-patients: A multicentre double-blind comparative trial. Br. J. Clin. Res..

[81] Kostiukova E.G., Granenov G.M., Andreĭchik L.A., Serditov O.V., Mosolov S.N. (2003). Comparative efficacy and tolerance of fluvoxamine and amitriptyline in the treatment of moderate and severe depression in mental hospital. Zh Nevrol Psikhiatr Im S S Korsakova.

[82] Remick R.A., Reesal R., Oakander M., Allen J., Claman J., Ramirez C.E., Perry K., Keller F.D. (1994). Comparison of fluvoxamine and amitriptyline in depressed outpatients. Curr. Ther. Res..

[83] Murasaki M., Mori A., Miura S. (1998). Clinical evaluation of SME3110 (fluvoxamine maleate) in the treatment of depression and depressive state: A double-blind, comparative study with amitriptyline. Rinsho-Iyaku (J. Clin. Ther. Med.).

[84] Mullin J.M., Pandita-Gunawardena V.R., Whitehead A.M. (1988). A double-blind comparison of fluvoxamine and dothiepin in the treatment of major affective disorder. Br. J. Clin. Pract..

[85] Rahman M.K., Akhtar M.J., Savla N.C., Sharma R.R., Kellett J.M., Ashford J.J. (1991). A double-blind, randomised comparison of fluvoxamine with dothiepin in the treatment of depression in elderly patients. Br. J. Clin. Pract..

[86] Tourigny-Rivard M., Nair N., Vincent P. Fluvoxamine versus desipramine in elderly patients with major depression: A double-blind comparison. Proceedings of the 9th ECNP (European College of Neuropsychopharmacology) Congress.

[87] Otsubo T., Akimoto Y., Yamada H., Koda R., Aoyama H., Tanaka K., Mimura M., Nakagome K., Kamijima K. (2005). A comparative study of the efficacy and safety profiles between fluvoxamine and nortriptyline in Japanese patients with major depression. Pharmacopsychiatry.

[88] Ansseau M., Gabriëls A., Loyens J., Bartholomé F., Evrard J.L., De Nayer A., Linhart R., Wirtz J., Bruynooghe F., Surinx K. (1994). Controlled comparison of paroxetine and fluvoxamine in major depression. Hum. Psychopharmacol. Clin. Exp..

[89] Kato M., Fukuda T., Wakeno M., Fukuda K., Okugawa G., Ikenaga Y., Yamashita M., Takekita Y., Nobuhara K., Azuma J. (2006). Effects of the serotonin type 2A, 3A and 3B receptor and the serotonin transporter genes on paroxetine and fluvoxamine efficacy and adverse drug reactions in depressed Japanese patients. Neuropsychobiology.

[90] Kiev A., Feiger A. (1997). A double-blind comparison of fluvoxamine and paroxetine in the treatment of depressed outpatients. J. Clin. Psychiatry.

[91] Nemeroff C.B., Ninan P.T., Ballenger J., Lydiard R.B., Feighner J., Patterson W.M., Greist J.H. (1995). Double-blind multicenter comparison of fluvoxamine versus sertraline in the treatment of depressed outpatients. Depression.

[92] Rossini D., Serretti A., Franchini L., Mandelli L., Smeraldi E., De Ronchi D., Zanardi R. (2005). Sertraline versus fluvoxamine in the treatment of elderly patients with major depression: A double-blind, randomized trial. J. Clin. Psychopharmacol..

[93] Haffmans P.M., Timmerman L., Hoogduin C.A. (1996). Efficacy and tolerability of citalopram in comparison with fluvoxamine in depressed outpatients: A double-blind, multicentre study. The LUCIFER Group. Int. Clin. Psychopharmacol..

[94] Dalery J., Honig A. (2003). Fluvoxamine versus fluoxetine in major depressive episode: A double-blind randomised comparison. Hum. Psychopharmacol..

[95] Rapaport M., Coccaro E., Sheline Y., Perse T., Holland P., Fabre L., Bradford D. (1996). A comparison of fluvoxamine and fluoxetine in the treatment of major depression. J. Clin. Psychopharmacol..

[96] Clerc G., Group M.F.S. (2001). Antidepressant efficacy and tolerability of milnacipran, a dual serotonin and noradrenaline reuptake inhibitor: A comparison with fluvoxamine. Int. Clin. Psychopharmacol..

[97] Ansseau M., von Frenckell R., Gérard M.-A., Mertens C., De Wilde J., Botte L., Devoitille J.-M., Evrard J.-L., De Nayer A., Darimont P. (1991). Interest of a loading dose of milnacipran in endogenous depressive inpatients: Comparison with the standard regimen and with fluvoxamine. Eur. Neuropsychopharmacol..

[98] Hackett D., Salinas E., Desmet A. (1998). Efficacy and safety of venlafaxine vs. fluvoxamine in outpatients with major depression. Eur. Neuropsychopharmacol..

[99] Schoemaker J., Gailledreau J., Hoyberg O.J. (2002). First, randomized, double-blind comparison of mirtazapine (15–45 mg) and fl uvoxamine (50–150 mg) in the treatment of depression. Int. J. Neuropsychopharmacol..

[100] Murasaki M., Schoemaker J.H., Miyake K., Gailledreau J., Heukels A.J., Fennema H.P., Sitsen J.M.A. (2010). Comparison of efficacy and safety of mirtazapine versus fluvoxamine in Japanese and Caucasian patients with major depressive disorder. Rinsho-Seishin-Yakuri (Jpn. J. Clin. Psychopharmacol.).

[101] Moon C.A., Jesinger D.K. (1991). The effects of psychomotor performance of fluvoxamine versus mianserin in depressed patients in general practice. Br. J. Clin. Pract..

[102] Perez A., Ashford J.J. (1990). A double-blind, randomized comparison of fluvoxamine with mianserin in depressive illness. Curr. Med. Res. Opin..

[103] Hieronymus F., Emilsson J.F., Nilsson S., Eriksson E. (2016). Consistent superiority of selective serotonin reuptake inhibitors over placebo in reducing depressed mood in patients with major depression. Mol. Psychiatry.

[104] Boschloo L., Hieronymus F., Lisinski A., Cuijpers P., Eriksson E. (2023). The complex clinical response to selective serotonin reuptake inhibitors in depression: A network perspective. Transl. Psychiatry.

[105] Westenberg H.G., Sandner C. (2006). Tolerability and safety of fluvoxamine and other antidepressants. Int. J. Clin. Pract..

[106] Thase M.E., Entsuah A.R., Rudolph R.L. (2001). Remission rates during treatment with venlafaxine or selective serotonin reuptake inhibitors. Br. J. Psychiatry.

[107] Smith D., Dempster C., Glanville J., Freemantle N., Anderson I. (2002). Efficacy and tolerability of venlafaxine compared with selective serotonin reuptake inhibitors and other antidepressants: A meta-analysis. Br. J. Psychiatry.

[108] de Silva V.A., Hanwella R. (2012). Efficacy and tolerability of venlafaxine versus specific serotonin reuptake inhibitors in treatment of major depressive disorder: A meta-analysis of published studies. Int. Clin. Psychopharmacol..

[109] Berlanga C., Flores-Ramos M. (2006). Different gender response to serotonergic and noradrenergic antidepressants. A comparative study of the efficacy of citalopram and reboxetine. J. Affect. Disord..

[110] Sramek J.J., Murphy M.F., Cutler N.R. (2016). Sex differences in the psychopharmacological treatment of depression. Dialogues Clin. Neurosci..

[111] Naito S., Sato K., Yoshida K., Higuchi H., Takahashi H., Kamata M., Ito K., Ohkubo T., Shimizu T. (2007). Gender differences in the clinical effects of fluvoxamine and milnacipran in Japanese major depressive patients. Psychiatry Clin. Neurosci..

[112] Hassanein E.H.M., Althagafy H.S., Baraka M.A., Abd-alhameed E.K., Ibrahim I.M. (2024). Pharmacological update of mirtazapine: A narrative literature review. Naunyn-Schmiedeberg’s Arch. Pharmacol..

[113] Anttila S.A., Leinonen E.V. (2001). A review of the pharmacological and clinical profile of mirtazapine. CNS Drug Rev..

[114] Van den Eynde V., Abdelmoemin W.R., Abraham M.M., Amsterdam J.D., Anderson I.M., Andrade C., Baker G.B., Beekman A.T.F., Berk M., Birkenhäger T.K. (2023). The prescriber’s guide to classic MAO inhibitors (phenelzine, tranylcypromine, isocarboxazid) for treatment-resistant depression. CNS Spectr..

[115] Van den Eynde V., Parker G., Ruhé H.G., Birkenhäger T.K., Godet L., Shorter E., Gillman P.K. (2023). On the Origins of MAOI Misconceptions: Reaffirming their Role in Melancholic Depression. Psychopharmacol. Bull..

[116] Athira K.V., Bandopadhyay S., Samudrala P.K., Naidu V.G.M., Lahkar M., Chakravarty S. (2020). An Overview of the Heterogeneity of Major Depressive Disorder: Current Knowledge and Future Prospective. Curr. Neuropharmacol..

[117] Buch A.M., Liston C. (2021). Dissecting diagnostic heterogeneity in depression by integrating neuroimaging and genetics. Neuropsychopharmacology.

[118] Li X., Yan D., Liao M., Zhang L., Li Z., Liu B., Chen Y., Zhang Y., Liu J., Li L. (2023). Effect of fluvoxamine on plasma interleukin-6 in patients with major depressive disorder: A prospective follow-up study. Front. Psychiatry.

[119] Liu P., Liu Z., Wang J., Gao M., Zhang Y., Yang C., Zhang A., Li G., Li X., Liu S. (2024). Immunoregulatory role of the gut microbiota in inflammatory depression. Nat. Commun..

[120] Mazza M.G., Zanardi R., Palladini M., Rovere-Querini P., Benedetti F. (2022). Rapid response to selective serotonin reuptake inhibitors in post-COVID depression. Eur. Neuropsychopharmacol..

[121] Smeraldi E., Zanardi R., Benedetti F., Di Bella D., Perez J., Catalano M. (1998). Polymorphism within the promoter of the serotonin transporter gene and antidepressant efficacy of fluvoxamine. Mol. Psychiatry.

[122] Yoshida K., Ito K., Sato K., Takahashi H., Kamata M., Higuchi H., Shimizu T., Itoh K., Inoue K., Tezuka T. (2002). Influence of the serotonin transporter gene-linked polymorphic region on the antidepressant response to fluvoxamine in Japanese depressed patients. Prog. Neuropsychopharmacol. Biol. Psychiatry.

[123] Yoshida K., Higuchi H., Kamata M., Takahashi H., Inoue K., Suzuki T., Itoh K., Ozaki N. (2007). The G196A polymorphism of the brain-derived neurotrophic factor gene and the antidepressant effect of milnacipran and fluvoxamine. J. Psychopharmacol..

[124] Kirchheiner J., Nickchen K., Bauer M., Wong M.L., Licinio J., Roots I., Brockmöller J. (2004). Pharmacogenetics of antidepressants and antipsychotics: The contribution of allelic variations to the phenotype of drug response. Mol. Psychiatry.

[125] Hicks J.K., Bishop J.R., Sangkuhl K., Müller D.J., Ji Y., Leckband S.G., Leeder J.S., Graham R.L., Chiulli D.L., LLerena A. (2015). Clinical Pharmacogenetics Implementation Consortium (CPIC) Guideline for CYP2D6 and CYP2C19 Genotypes and Dosing of Selective Serotonin Reuptake Inhibitors. Clin. Pharmacol. Ther..

[126] Zastrozhin M.S., Grishina E.A., Denisenko N.P., Skryabin V.Y., Markov D.D., Savchenko L.M., Bryun E.A., Sychev D.A. (2018). Effects of CYP2D6 genetic polymorphisms on the efficacy and safety of fluvoxamine in patients with depressive disorder and comorbid alcohol use disorder. Pharmacogenomics Pers. Med..

[127] Carrillo J.A., Dahl M.L., Svensson J.O., Alm C., Rodríguez I., Bertilsson L. (1996). Disposition of fluvoxamine in humans is determined by the polymorphic CYP2D6 and also by the CYP1A2 activity. Clin. Pharmacol. Ther..

[128] Ji L.L., Peng J.B., Fu C.H., Cao D., Li D., Tong L., Wang Z.Y. (2016). Activation of Sigma-1 receptor ameliorates anxiety-like behavior and cognitive impairments in a rat model of post-traumatic stress disorder. Behav. Brain Res..

[129] Toyohara J., Sakata M., Ishiwata K. (2012). Roles of σ1 receptors in the mechanisms of action of CNS drugs. Transl. Neurosci..

[130] Sałaciak K., Pytka K. (2022). Revisiting the sigma-1 receptor as a biological target to treat affective and cognitive disorders. Neurosci. Biobehav. Rev..

[131] https://www.crd.york.ac.uk/prospero/.

[132] Pollock M.F.R., Becker L.A., Pieper D., Hartling L., Higgins J.P.T., Thomas J., Chandler J., Cumpston M., Li T., Page M.J., Welch V.A. (2023). Chapter V: Overviews of Reviews. Cochrane Handbook for Systematic Reviews of Interventions.

[133] Lin L., Aloe A.M. (2021). Evaluation of various estimators for standardized mean difference in meta-analysis. Stat. Med..

[134] Shea B.J., Reeves B.C., Wells G., Thuku M., Hamel C., Moran J., Moher D., Tugwell P., Welch V., Kristjansson E. (2017). AMSTAR 2: A critical appraisal tool for systematic reviews that include randomised or non-randomised studies of healthcare interventions, or both. BMJ.

[135] Stein D.J., Westenberg H.G., Yang H., Li D., Barbato L.M. (2003). Fluvoxamine CR in the long-term treatment of social anxiety disorder: The 12- to 24-week extension phase of a multicentre, randomized, placebo-controlled trial. Int. J. Neuropsychopharmacol..

[136] Hudson J.I., McElroy S.L., Raymond N.C., Crow S., Keck P.E., Carter W.P., Mitchell J.E., Strakowski S.M., Pope H.G., Coleman B.S. (1998). Fluvoxamine in the treatment of binge-eating disorder: A multicenter placebo-controlled, double-blind trial. Am. J. Psychiatry.

[137] Amore M., Bellini M., Berardi D., Berlinzani L., Cervino G., Cremonini A., Ferrari G., Innamorati A. (1989). Double-blind comparison of fluvoxamine and imipramine in depressed patients. Curr. Ther. Res..

[138] De Wilde J.E., Doogan D.P. (1982). Fluvoxamine and chlorimipramine in endogenous depression. J. Affect. Disord..

[139] Dick P., Ferrero E. (1983). A double-blind comparative study of the clinical efficacy of fluvoxamine and chlorimipramine. Br. J. Clin. Pharmacol..

[140] Gonella G., Baignoli G., Ecari U. (1990). Fluvoxamine and imipramine in the treatment of depressive patients: A double-blind controlled study. Curr. Med. Res. Opin..

[141] Guelfi J.D., Dreyfus J.F., Pichot P. (1983). A double-blind controlled clinical trial comparing fluvoxamine with imipramine. Br. J. Clin. Pharmacol..

[142] Klok C.J., Brouwer G.J., van Praag H.M., Doogan D. (1981). Fluvoxamine and clomipramine in depressed patients. A double-blind clinical study. Acta Psychiatr. Scand..

[143] Nathan R.S., Perel J.M., Pollock B.G., Kupfer D.J. (1990). The role of neuropharmacologic selectivity in antidepressant action: Fluvoxamine versus desipramine. J. Clin. Psychiatry.

[144] Gasperini M., Gatti F., Bellini L., Anniverno R., Smeraldi E. (1992). Perspectives in clinical psychopharmacology of amitriptyline and fluvoxamine. A double-blind study in depressed inpatients. Neuropsychobiology.

[145] Kasper S., Voll G., Vieira A., Kick H. (1990). Response to total sleep deprivation before and during treatment with fluvoxamine or maprotiline in patients with major depression-results of a double-blind study. Pharmacopsychiatry.

[146] Conti, Placidi G.F., Dell L., Lenzi A., Cassano G.B. (1987). Therapeutic response in subtypes of major depression. New Trends Exp. Clin. Psychiatry.

[147] Pöldinger W., Bures E. (1984). Fluvoxamine in patients with depressive disorder. Proceedings of the International Symposium on Fluvoxamine.

[148] Wagner, Wakelin J., Coleman B.S., Cimander K. (1985). Therapeutische Ergebnisse mit Fluvoxamin und der Einfluß psychotroper Begleitmedikation auf Wirksamkeit und Verträglichkeit. Adv. Pharrnacother..

[149] Wakelin J.S. (1986). Fluvoxamine in the treatment of the older depressed patient; double-blind, placebo-controlled data. Int. Clin. Psychopharmacol..

[150] de Jonghe F., Swinkels J., Tuynman-Qua H. (1991). Randomized double-blind study of fluvoxamine and maprotiline in treatment of depression. Pharmacopsychiatry.

[151] Bramanti P., Ricci R.M., Roncari R., Bilone F., Inga F., Teti V., DeCristofaro A.M., Ceccarelli G., DiPerri R., Candela L. (1988). An Italian multicentre experience with fluvoxamine, a new antidepressant drug, versus imipramine. Curr. Ther. Res..

