# Supplementary material for: An Overview of the Systematic Reviews About the Efficacy of Fluvoxamine on Depression
Source: Pharmaceuticals (Basel). 2025 May 12;18(5):711. doi: 10.3390/ph18050711 (PMC12115031; doi:10.3390/ph18050711)
Supplement: Supplementary file 1 [file pharmaceuticals-18-00711-s001.zip › pharmaceuticals-3577805-supplementary.pdf]

AN OVERVIEW OF THE SYSTEMATIC REVIEWS ABOUT THE EFFICACY OF FLUVOXAMINE ON DEPRESSION

SUPPLEMENTARY MATERIAL

Michel Haddad, Luiz Henrique Junqueira Dieckmann, Thiago Wendt Viola, Franciele Franco Scarante, Naielly Rodrigues da Silva, Jair de Jesus Mari

Table S1. PRISMA Checklist. The PRISMA Checklist can be found at http://prisma-statement.org/.

Section and Topic	Item #	Checklist item	Reported on page
**TITLE**			
Title	1	Identify the report as a systematic review.	1
**ABSTRACT**			
Abstract	2	See the PRISMA 2020 for Abstracts checklist.	1
**INTRODUCTION**			
Rationale	3	Describe the rationale for the review in the context of existing knowledge.	2-3
Objectives	4	Provide an explicit statement of the objective(s) or question(s) the review addresses.	3
**METHODS**			
Eligibility criteria	5	Specify the inclusion and exclusion criteria for the review and how studies were grouped for the syntheses.	17-18
Information sources	6	Specify all databases, registers, websites, organizations, reference lists and other sources searched or consulted to identify studies. Specify the date when each source was last searched or consulted.	4 and 17-18
Search strategy	7	Present the full search strategies for all databases, registers and websites, including any filters and limits used.	17
Selection process	8	Specify the methods used to decide whether a study met the inclusion criteria of the review, including how many reviewers screened each record and each report retrieved, whether they worked independently, and if applicable, details of automation tools used in the process.	18
Data collection process	9	Specify the methods used to collect data from reports, including how many reviewers collected data from each report, whether they worked independently, any processes for obtaining or confirming data from study investigators, and if applicable, details of automation tools used in the process.	18
Data items	10a	List and define all outcomes for which data were sought. Specify whether all results that were compatible with each outcome domain in each study were sought (e.g. for all measures, time points, analyses), and if not, the methods used to decide which results to collect.	18
	10b	List and define all other variables for which data were sought (e.g. participant and intervention characteristics, funding sources). Describe any assumptions made about any missing or unclear information.	18
Study risk of bias assessment	11	Specify the methods used to assess risk of bias in the included studies, including details of the tool(s) used, how many reviewers assessed each study and whether they worked independently, and if applicable, details of automation tools used in the process.	18
Effect measures	12	Specify for each outcome the effect measure(s) (e.g. risk ratio, mean difference) used in the synthesis or presentation of results.	18
Synthesis	13a	Describe the processes used to decide which studies were eligible for each synthesis (e.g. tabulating the study intervention	18-19

Section and Topic	Item #	Checklist item	Reported on page
methods		characteristics and comparing against the planned groups for each synthesis (item #5)).	
	13b	Describe any methods required to prepare the data for presentation or synthesis, such as handling of missing summary statistics, or data conversions.	18-19
	13c	Describe any methods used to tabulate or visually display results of individual studies and syntheses.	18-19
	13d	Describe any methods used to synthesize results and provide a rationale for the choice(s). If meta-analysis was performed, describe the model(s), method(s) to identify the presence and extent of statistical heterogeneity, and software package(s) used.	18-19
	13e	Describe any methods used to explore possible causes of heterogeneity among study results (e.g. subgroup analysis, meta-regression).	18
	13f	Describe any sensitivity analyses conducted to assess robustness of the synthesized results.	19
Reporting bias assessment	14	Describe any methods used to assess risk of bias due to missing results in a synthesis (arising from reporting biases).	18
Certainty assessment	15	Describe any methods used to assess certainty (or confidence) in the body of evidence for an outcome.	18-19
**RESULTS**			
Study selection	16a	Describe the results of the search and selection process, from the number of records identified in the search to the number of studies included in the review, ideally using a flow diagram.	3-4, Figure 1
	16b	Cite studies that might appear to meet the inclusion criteria, but which were excluded, and explain why they were excluded.	Figure 1
Study characteristics	17	Cite each included study and present its characteristics.	3-13, Tables 1, 2 and S4
Risk of bias in studies	18	Present assessments of risk of bias for each included study.	Table S4
Results of individual studies	19	For all outcomes, present, for each study: (a) summary statistics for each group (where appropriate) and (b) an effect estimate and its precision (e.g. confidence/credible interval), ideally using structured tables or plots.	Tables 1, 2 and S4
Results of syntheses	20a	For each synthesis, briefly summarize the characteristics and risk of bias among contributing studies.	Tables S4
	20b	Present results of all statistical syntheses conducted. If meta-analysis was done, present for each the summary estimate and its precision (e.g. confidence/credible interval) and measures of statistical heterogeneity. If comparing groups, describe the direction of the effect.	Tables 1, 2 and S3
	20c	Present results of all investigations of possible causes of heterogeneity among study results.	3-13
	20d	Present results of all sensitivity analyses conducted to assess the robustness of the synthesized results.	Tables 1, 2 and S4
Reporting biases	21	Present assessments of risk of bias due to missing results (arising from reporting biases) for each synthesis assessed.	Table S4
Certainty of	22	Present assessments of certainty (or confidence) in the body of evidence for each outcome assessed.	Table S4

Section and Topic	Item #	Checklist item	Reported on page
evidence			
**DISCUSSION**			
Discussion	23a	Provide a general interpretation of the results in the context of other evidence.	14
	23b	Discuss any limitations of the evidence included in the review.	16
	23c	Discuss any limitations of the review processes used.	16
	23d	Discuss implications of the results for practice, policy, and future research.	14-16
**OTHER INFORMATION**			
Registration and protocol	24a	Provide registration information for the review, including register name and registration number, or state that the review was not registered.	17
	24b	Indicate where the review protocol can be accessed, or state that a protocol was not prepared.	17
	24c	Describe and explain any amendments to information provided at registration or in the protocol.	17
Support	25	Describe sources of financial or non-financial support for the review, and the role of the funders or sponsors in the review.	19
Competing interests	26	Declare any competing interests of review authors.	19
Availability of data, code and other materials	27	Report which of the following are publicly available and where they can be found: template data collection forms; data extracted from included studies; data used for all analyses; analytic code; any other materials used in the review.	Table S4

Table S2. Assessment of methodological quality of the included systematic reviews using the tool "A Measurement Tool to Assess Systematic Reviews, version 2" (AMSTAR-2) [1].

First Author	Year of publication	1	2	3	4	5	6	7	8	9	10	11	12	13	14	15	16	Total score	Qualitative score
Yuan	2020	0	0	1	1	1	1	1	1	1	0	1	1	0	1	1	1	12	Moderate
Cipriani	2018	1	1	1	1	1	1	1	1	1	1	1	1	1	1	1	1	16	High
Omori	2014	1	1	1	1	1	1	1	1	1	1	1	1	1	1	1	1	16	High
Nakagawa	2009	1	1	1	1	1	0	1	1	1	1	1	1	1	1	1	1	15	High
Omori	2009	1	0	1	1	0	1	1	1	1	1	1	1	1	1	1	1	14	High
Ramsberg	2012	1	0	1	1	0	1	1	1	0	0	1	0	1	1	0	1	10	Moderate
Kishi	2023	1	1	1	1	1	1	1	1	1	1	1	1	1	1	1	1	16	High
Suchting	2021	1	0	1	1	1	1	1	1	1	0	1	1	1	1	0	1	13	Moderate
Anderson	1994	1	0	1	1	0	0	1	0	0	0	1	0	1	1	1	0	8	Low
Moller	1994	1	0	1	0	0	0	0	1	0	0	1	0	0	0	0	0	4	Low
Lopez-Ibor	1996	1	0	0	0	0	0	0	1	0	0	1	0	0	0	0	0	3	Low
Anderson	2000	1	0	1	0	0	0	0	1	0	0	1	0	0	1	1	0	6	Moderate
Nemeroff	2008	1	0	1	1	0	0	1	1	1	1	1	1	1	1	1	1	13	Moderate
Cipriani	2009	1	1	1	1	1	1	1	1	1	1	1	1	1	1	0	1	15	High

Table S3. Items and questions of the AMSTAR-2 used on the **a**ssessment of methodological quality of the included systematic reviews.

**Items and Questions**
1. Did the research questions and inclusion criteria for the review include the components of PIC?
2. Did the report of the review contain an explicit statement that the review methods were established prior to the conduct of the review, and did the report justify any significant deviations from the protocol?
3. Did the review authors explain their selection of the study designs for inclusion in the review?
4. Did the review authors use a comprehensive literature search strategy?
5. Did the review authors perform study selection in duplicate?
6. Did the review authors perform data extraction in duplicate?
7. Did the review authors provide a list of excluded studies and justify the exclusions?
8. Did the review authors describe the included studies in adequate detail?
9. Did the review authors use a satisfactory technique for assessing the risk of bias in individual studies that were included in the review?
10. Did the review authors report on the sources of funding for the studies included in the review?
11. If meta-analysis was performed, did the review authors use appropriate methods for statistical combination of results?
12. If meta-analysis was performed, did the review authors assess the potential impact of RoB in individual studies on the results of the meta-analysis or other evidence synthesis?
13. Did the review authors account for risk of bias in individual studies when interpreting/discussing the results of the review?
14. Did the review authors provide a satisfactory explanation for, and discussion of, any heterogeneity observed in the results of the review?
15. If they performed quantitative synthesis, did the review authors carry out an adequate investigation of publication bias (small study bias) and discuss its likely impact on the results of the review?
16. Did the review authors report any potential sources of conflict of interest, including any funding they received for conducting the review?

Figure S1. Pearson's Correlation between AMSTAR-2 scores and publication year of the included reviews. A two-tailed p-value < 0.05 was considered statistically significant.

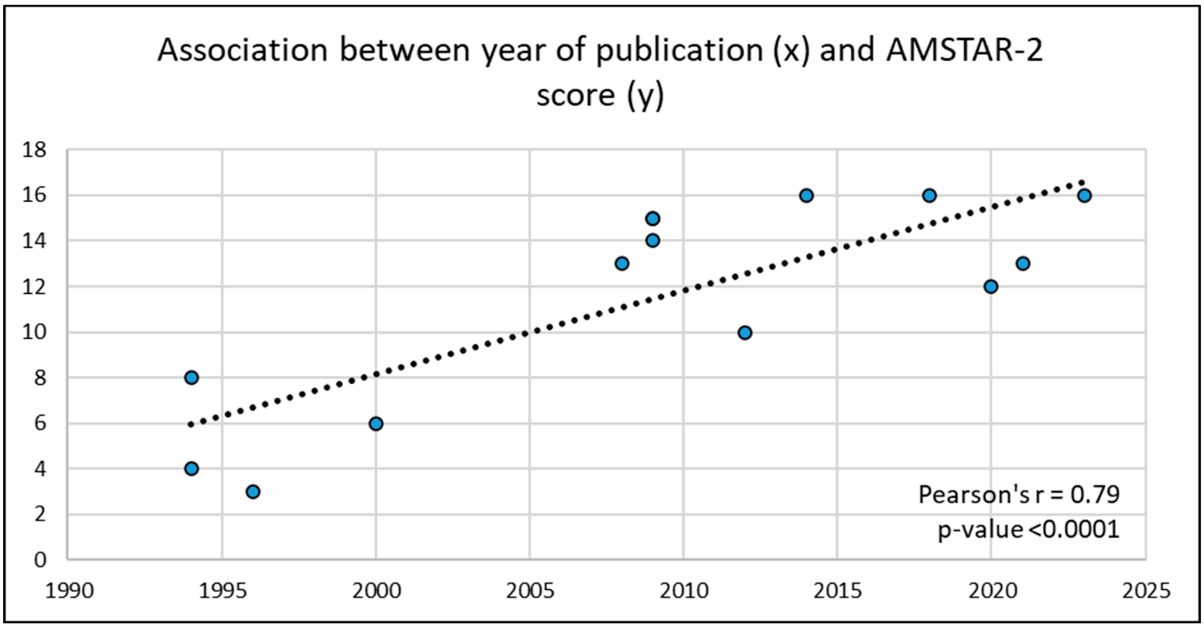

Table S4. Comprehensive summary of the overview of reviews, encompassing all findings from the included reviews, independently of the AMSTAR classification. Given the large number of comparisons presented in NMAs, we have chosen to include only direct comparisons. The characteristics included systematic reviews and meta-analyses on the efficacy of fluvoxamine in the treatment of depression. Response was defined as 50% improvement from baseline to end point on depression rating scales or who scored much improved or very much improved on the clinical global impression (CGI) scale, and remission was defined as number of patients showing scores ≤7 on Hamilton Depression Rating Scale (HRSD) 17 items, ≤8 on for all the other longer versions of HRSD, and/or ≤11 on Montgomery–Åsberg Depression Rating Scale (MADRS) or patients who were "not ill or borderline mentally ill" (score 1 or 2) on the CGI-Severity score out of the total number of randomized patients. Abbreviations: CCA – corrected covered area regarding RCTs (6%–10% is considered "moderate overlap," 11%–15% is considered "high overlap," and >15% is considered "very high overlap"); CI, confidence interval; db, double-blinded; ES, effect size; No, sample size; NK, not known; NMA, network meta-analysis; MA, meta-analysis; OR, odds ratio; RBR, relative benefit ratio; RCT, randomized clinical trial; RD, rate difference; RES, relative effect size; ol, open label; TCA, tricyclic antidepressants; UR, umbrella review. The umbrella review was not included in the CCA analysis given that it included individual reviews and not RCTs. Specific information about the RCTs included in the remission analysis by Cipriani et al. (2018) was not reported. Remission-related outcomes are highlighted in light orange. *These studies were considered to originate from the same dataset reported by Hackett et al., 1998 [66].

Review	Source	Included studies No (Patients, No)	Time of follow-up (weeks)	Blinding RCTs	No patients per arm (fluvoxamine, comparator)	Measure of efficacy	Summary estimates (ES, 95% CI)	Main findings	AMSTAR
**Fluvoxamine** ***vs*** **Placebo**<br>*Primary studies overlap = 10% (moderate overlap)*									
Yuan et al., 2020 [2]	MA	Hudson et al., 1998 [3] (85)	9	db	42,43	Response	OR = 1.97 (1.00, 3.89)	Fluvoxamine significantly more effective than placebo	Moderate
		Stein et al., 2003 [4] (109)	24	db	56, 53				
Yuan et al., 2020 [2]	MA	Hudson et al., 1998 [3] (85)	9	db	42,43	Remission	OR = 1.86 (0.98, 3,52)	Fluvoxamine significantly more effective than placebo	Moderate
		Stein et al., 2003 [4] (109)	24	db	56, 53				
Anderson and	MA	Amin et al., 1984 [6] (311)	4	db	161, 150	Reduction in HAMD score	ES = 0.34 (0.15, 0.52)	Fluvoxamine significantly more effective than placebo	Low
		Feighner et al., 1989 [7] (33)	6	db	21, 12				
		Lydiard et al., 1989 [8] (36)	6	db	18, 18				

Tomenson, 1994 [5]		March et al., 1990 [9] (36)	6	db	18, 18				
		Roth et al., 1990 [10] (60)	6	db	30, 30				
Kishi et al., 2023 [11]	NMA	Terra & Montogomery, 1998 [12] (204)	52	db	110, 94	6-month relapse rate	RR = 0.298 (0.114, 0.686)	Fluvoxamine significantly more effective than placebo	High
Cipriani et al., 2018 [13]	NMA	Amin et al., 1984 [6] (311)	4	db	161, 150	Response	OR = 1.69 (1.41, 2.02)	Fluvoxamine significantly more effective than placebo	High
		Brown et al., 1986 [14] (64)	6	db	33, 31				
		Cassano et al., 1986 [15] (310)	4	db	161, 149				
		Claghorn et al., 1996 [16] (100)	6	db	50, 50				
		Dominguez et al., 1985 [17] (66)	4	db	35, 31				
		Fabre et al., 1996 [18] (100)	6	db	50, 50				
		Feighner et al., 1989b [7] (50)	6	db	31, 19				
		Itil et al., 1983 [19] (44)	4	db	22, 22				
		Lapierre et al., 1987 [20] (42)	6	db	22, 20				
		Lydiard et al. 1989 [8] (36)	6	db	18, 18				
		March et al., 1990 [9] (36)	6	db	18, 18				
		Norton et al., 1984 [21] (60)	4	db	35, 25				
		Roth et al., 1990 [10] (60)	6	db	30, 30				
		Walczak et al., 1996 [22] (500)	6	db	300, 200				
Cipriani et al., 2018 [13]	NMA	Amin et al., 1984 [6] (311)	4	db	161, 150	Remission	OR = 0.58 (0.39, 0.86)	Fluvoxamine significantly more effective than placebo	High
		Brown et al., 1986 [14] (64)	6	db	33, 31				
		Cassano et al., 1986 [15] (310)	4	db	161, 149				
		Claghorn et al., 1996 [16] (100)	6	db	50, 50				
		Dominguez et al., 1985 [17] (66)	4	db	35, 31				
		Fabre et al., 1996 [18] (100)	6	db	50, 50				
		Feighner et al., 1989b [7] (50)	6	db	31, 19				
		Itil et al., 1983 [19] (44)	4	db	22, 22				
		Lapierre et al., 1987 [20] (42)	6	db	22, 20				

		Lydiard et al., 1989 [8] (36)	6	db	18, 18				
		March et al., 1990 [9] (36)	6	db	18, 18				
		Norton et al., 1984 [21] (60)	4	db	35, 25				
		Roth et al., 1990 [10] (60)	6	db	30, 30				
		Walczak et al., 1996 [22] (500)	6	db	300, 200				
**Fluvoxamine vs TCA**									
**Unspecified TCA**<br>*Primary studies overlap = 61% (very high overlap)*									
Anderson and Tomenson, 1994 [5]	MA	Amin et al. 1984 [6] (314)	4-6	db	161, 153	Reduction in HAMD score	ES = 0.00 (-0.13, 0.12)	Non-significant difference	Low
		Amore et al., 1989 [23] (30)	4	db	15, 15				
		de Wilde & Doogan, 1982 [24] (30)	4	db	15, 15				
		de Wilde et al., 1983 [25] (43)	6	db	21, 23				
		Dick & Ferrero, 1983 [26] (32)	4	db	17, 15				
		Feighner et al., 1989 [7] (48)	6	db	21, 27				
		Gonella et al., 1990 [27] (20)	4	db	10, 10				
		Guelfi et al., 1983 [28] (127)	4	db	59, 68				
		Guy et al., 1991 [29] (18)	4-6	db	10, 8				
		Harris et al., 1991 [30] (69)	6	db	35, 34				
		Klok et al., 1981 [31] (28)	4	db	13, 15				
		Lydiard et al., 1989 [8] (36)	6	db	18, 18				
		March et al., 1990 [9] (36)	6	db	18, 18				
		Mullin et al., 1989 [32] (50)	6	db	26, 24				
		Nathan et al., 1990 [33] (40)	4	db	20, 20				
		Rahman et al., 1991 [34] (36)	6	db	17, 19				
		Roth et al., 1990 [10] (51)	6	db	27, 24				
Anderson et al., 2000 [35]	MA	Amin et al. 1984 [6] (314)	4-6	db	161, 153	Reduction in HAMD or MADRS score	RES = -0.01 (-0.11, 0.1)	Non-significant difference	Moderate
		Amore et al., 1989 [23] (25)	4	db	15, 10				
		Bramanti et al., 1988 (57)	4	db	28, 29				
		Claghorn et al., 1996 (88)	6	db	44, 44				
		de Jonghe et al., 1991[a] (42)	2	db	21, 21				
		de Wilde & Doogan, 1982 [24] (30)	4	db	15, 15				

		de Wilde et al., 1983 [25] (43)	6	db	21, 23				
		Dick & Ferrero, 1983 [26] (32)	4	db	16, 13				
		Dominguez et al., 1985 [17] (70)	6	db	35, 35				
		Fabre et al., 1996 [18] (100)	6	db	50,50				
		Feighner et al., 1989 [7] (48)	6	db	21, 27				
		Gasperini et al., 1992 [36] (56)	6	db	30, 26				
		Gonella et al., 1990 [27] (20)	4	db	10, 10				
		Guelfi et al., 1983 [28] (127)	4	db	59, 68				
		Guy et al., 1991 [29] (18)	4-6	db	10, 8				
		Harris et al., 1991 [30] (69)	6	db	35, 34				
		Itil et al., 1983 [19] (47)	6	db	22, 25				
		Kasper et al., 1990 [37] (41)	2	db	21,20				
		Klok et al., 1981 [31] (28)	4	db	13, 15				
		Lapierre et al., 1987 [20] (43)	2	db	22,21				
		Lydiard et al., 1989 [8] (36)	6	db	18, 18				
		March et al., 1990 [9] (36)	6	db	13,15				
		Mullin et al., 1988 [32] (50)	6	db	26, 24				
		Nathan et al., 1990 [33] (40)	4	db	17,18				
		Norton et al., 1984 [21] (66)	6	db	35,31				
		Ottevanger, 1995 [38] (40)	6	db	20, 20				
		Rahman et al., 1991 [34] (36)	6	db	17, 19				
		Remick et al., 1994 [39] (22)	2	db	13, 9				
		Roth et al., 1990 [10] (51)	6	db	27, 24				
Omori et al., 2010 [40]	MA	Claghorn et al., 1996 [16] (100)	6	db	50,50	Response	OR = 0.97 (0.73, 1.29)	Non-significant difference	High
		Fabre et al., 1996 [18] (100)	6	db	50,50				
		Feighner et al., 1989 [7] (67)	6	db	31,36				
		Guy et al., 1984 [29] (36)	6	db	17,19				
		Lydiard et al., 1989 [8] (36)	6	db	18,18				
		March et al., 1990 [9] (36)	6	db	18,18				
		de Wilde et al., 1983 [25] (73)	6	db	22, 51				
		Zohar et al., 2003 [41] (86)	8	db	44, 42				

		Barge-Schaapveld et al., 1995 [42] (23)	6	ol	13, 10				
		Harris et al., 1991 [30] (69)	6	db	35, 34				
		Kostiukova et al., 2003 [43] (60)	6	ol	30, 30				
		Remick et al., 1994 [39] (33)	7	db	16, 17				
		Otsubo et al., 2005 [44] (74)	8	ol	36, 38				
		Mullin et al., 1988 [32] (73)	6	db	37, 36				
		Rahman et al., 1991 [34] (52)	6	db	26, 26				
		Tourigny-Rivard et al., 1996 [45] (47)	10	db	22, 25				
Omori et al., 2010 [40]	MA	Claghorn et al., 1996 [16] (100)	6	db	50,50	Remission	OR = 1.00 (0.69, 1.45)	Non-significant difference	High
		Fabre et al., 1996 [18] (100)	6	db	50,50				
		Feighner et al., 1989 [7] (67)	6	db	31,36				
		Guy et al., 1984 [29] (36)	6	db	17,19				
		Lydiard et al., 1989 [8] (36)	6	db	18,18				
		March et al., 1990 [9] (36)	6	db	18,18				
		de Wilde et al., 1983 [25] (73)	6	db	22, 51				
		Zohar et al., 2003 [41] (86)	8	db	44, 42				
		Barge-Schaapveld et al., 1995 [42] (23)	6	ol	13, 10				
		Harris et al., 1991 [30] (69)	6	db	35, 34				
		Kostiukova et al., 2003 [43] (60)	6	ol	30, 30				
		Remick et al., 1994 [39] (33)	7	db	16, 17				
		Otsubo et al., 2005 [44] (74)	8	ol	36, 38				
		Mullin et al., 1988 [32] (73)	6	db	37, 36				
		Rahman et al., 1991 [34] (52)	6	db	26, 26				
		Tourigny-Rivard et al., 1996 [45] (47)	10	db	22, 25				
Omori et al., 2009 [46]	MA	Brown et al., 1986 [14] (50)	6	db	33,17	Response	RR = 0.99 (0.86, 1.14)	Non-significant difference	High
		Claghorn et al., 1996 [16] (100)	6	db	50,50				
		Guy et al., 1984 [29] (36)	6	db	17,19				

		Lydiard et al., 1989 [8] (36)	6	db	18,18				
		March et al., 1990 [9] (36)	6	db	18,18				
		Miller et al., 2001 [47] (24)	6	db	13,11				
		de Wilde et al., 1983 [25] (73)	6	db	22, 51				
		Zohar et al., 2003 [41] (86)	8	db	44, 42				
		Barge-Schaapveld et al., 1995 [42] (23)	6	ol	13, 10				
		Harris et al., 1991 [30] (69)	6	db	35, 34				
		Kostiukova et al., 2003 [43] (60)	6	ol	30, 30				
		Remick et al., 1994 [39] (33)	7	db	16, 17				
		Mullin et al., 1988 [32] (73)	6	db	37, 36				
		Rahman et al., 1991 [34] (52)	6	db	26, 26				
		Otsubo et al., 2005 [44] (74)	8	ol	36, 38				
		Tourigny-Rivard et al., 1996 [45] (47)	10	db	22,25				
Omori et al., 2009 [46]	MA	Brown et al., 1986 [14] (50)	6	db	33,17	Remission	RR = 0.98 (0.71, 1.35)	Non-significant difference	High
		Claghorn et al., 1996 [16] (100)	6	db	50,50				
		Guy et al., 1984 [29] (36)	6	db	17,19				
		Lydiard et al., 1989 [8] (36)	6	db	18,18				
		March et al., 1990 [9] (36)	6	db	18,18				
		Miller et al., 2001 [47] (24)	6	db	13,11				
		de Wilde et al., 1983 [25] (73)	6	db	22, 51				
		Zohar et al., 2003 [41] (86)	8	db	44, 42				
		Barge-Schaapveld et al., 1995 [42] (23)	6	ol	13, 10				
		Harris et al., 1991 [30] (69)	6	db	35, 34				
		Kostiukova et al., 2003 [43] (60)	6	ol	30, 30				
		Remick et al., 1994 [39] (33)	7	db	16, 17				
		Mullin et al., 1988 [32] (73)	6	db	37, 36				
		Rahman et al., 1991 [34] (52)	6	db	26, 26				
		Otsubo et al., 2005 [44] (74)	8	ol	36, 38				

		Tourigny-Rivard et al., 1996 [45](47)	10	db	22,25				
**Imipramine**<br>*Primary studies overlap = 12% (high overlap)*									
Omori et al., 2010 [40]	MA	Claghorn et al., 1996 [16] (100)	6	db	50,50	Response	OR = 0.97 (0.59, 1,58)	Non-significant difference	High
		Fabre et al., 1996 [18] (100)	6	db	50,50				
		Feighner et al., 1989 [7] (67)	6	db	31,36				
		Guy et al., 1984 [29] (36)	6	db	17,19				
		Itil et al., 1983 [19] (47)	6	db	22,25				
		Lydiard et al., 1989 [8] (36)	6	db	18,18				
		March et al., 1990 [9] (36)	6	db	18,18				
Omori et al., 2010 [40]	MA	Claghorn et al., 1996 [16] (100)	6	db	50,50	Remission	OR = 1.07 (0.59, 1.94)	Non-significant difference	High
		Fabre et al., 1996 [18] (100)	6	db	50,50				
		Feighner et al., 1989 [7] (67)	6	db	31,36				
		Guy et al., 1984 [29] (36)	6	db	17,19				
		Lydiard et al., 1989 [8] (36)	6	db	18,18				
		March et al., 1990 [9] (36)	6	db	18,18				
Omori et al., 2009 [46]	MA	Brown et al., 1986 [14] (50)	6	db	33,17	Response	RR = 0.95 (0.67, 1.36)	Non-significant difference	High
		Claghorn et al., 1996 [16] (100)	6	db	50,50				
		Guy et al., 1984 [29] (36)	6	db	17,19				
		Lydiard et al., 1989 [8] (36)	6	db	18,18				
		March et al., 1990 [9] (36)	6	db	18,18				
		Miller et al., 2001 [47] (24)	6	db	13,11				
Omori et al., 2009 [46]	MA	Brown et al., 1986 [14] (50)	6	db	33,17	Remission	RR = 1.03 (0.53, 2.00)	Non-significant difference	High
		Claghorn et al., 1996 [16] (100)	6	db	50,50				
		Guy et al., 1984 [29] (36)	6	db	17,19				
		Lydiard et al., 1989 [8] (36)	6	db	18,18				
		March et al., 1990 [9] (36)	6	db	18,18				
		Miller et al., 2001 [47] (24)	6	db	13,11				

Ramsberg et al., 2012 [48]	NMA	Birkenhäger et al., 2004 [49](138)	6	db	68, 70	Probability of remission	OR = 1.4 (0.872, 2.133)	Non-significant difference	Moderate
Möller et al., 1994 [50]	MA	Amin et al., 1984 [6] (314)	4-6	db	161, 153	Reduction HAMD and psychometric measures such as the CGI scale	ES = -0.12(-0.41, 0.05)	Non-significant difference	Low
		Conti et al., 1987 [51] (202)	4-6	db	101,101				
		Dominguez et al., 1985 [17] (70)	6	db	35, 35				
		Feighner et al., 1989 [7] (67)	6	db	31,36				
		Guelfi et al., 1983 [28] (127)	4	db	59, 68				
		Guy et al., 1984 [29] (36)	6	db	17,19				
		Itil et al., 1983 [19] (47)	6	db	22,25				
		Lapierre et al., 1987 [20] (43)	2	db	22,21				
		Norton et al., 1984 [21] (66)	6	db	35,31				
		Pöldinger and Bures, 1984 [52](20)	4	db	10,10				
		Wagner et al., 1985 [53] (481)	4-6	db	N.K.				
		Wakelin, 1986 [54] (76)	4	db	29, 47				
**Amitriptyline or Clomipramine**									
Anderson and Tomenson , 1994 [5]	MA	de Wilde & Doogan, 1982 [24] (30)	4	db	15, 15	Reduction in HAMD score or MADRS	ES = -0.16 -(0.46, 0.13)	Non-significant difference	Low
		de Wilde et al., 1983 [25] (43)	6	db	21, 23				
		Dick & Ferrero, 1983 [26] (32)	4	db	17, 15				
		Harris et al., 1991 [30] (69)	6	db	35, 34				
		Klok et al., 1981 [31] (28)	4	db	13, 15				
**Clomipramine**<br>*Primary studies overlap = 13% (high overlap)*									
Omori et al., 2010 [40]	MA	de Wilde et al., 1983 [25] (73)	6	db	22, 51	Response	OR = 0.84 (0.38, 1.85)	Non-significant difference	High
		Zohar et al., 2003 [41] (86)	8	db	44, 42				
Omori et al., 2010 [40]	MA	de Wilde et al., 1983 [25] (73)	6	db	22, 51	Remission	OR = 0.64 (0.28, 1.49)	Non-significant difference	High
		Zohar et al., 2003 [41] (86)	8	db	44, 42				

Omori et al., 2009 [46]	MA	Zohar et al., 2003 [41] (86)	8	db	44, 42	Response	RR = 0.99 (0.68, 1.44)	Non-significant difference	High
Omori et al., 2009 [46]	MA	Zohar et al., 2003 [41] (86)	8	db	44, 42	Remission	RR = 0.72 (0.20, 2.56)	Non-significant difference	High
Cipriani et al., 2018 [13]	NMA	De Wilde et al., 1983 [25] (43)	6	db	22, 21	Response	OR = 1.01 (0.76, 1.32)	Non-significant difference	High
		Ottevanger, 1995 [38] (40)	4	db	20, 20				
Cipriani et al., 2018 [13]	NMA	De Wilde et al., 1983 [25] (43)	6	db	22, 21	Remission	OR = 1.57 (0.56, 4.57)	Non-significant difference	High
		Ottevanger, 1995 [38] (40)	4	db	20, 20				
**Amitriptyline**<br>*Primary studies overlap = 60% (very high overlap)*									
Omori et al., 2010 [40]	MA	Barge-Schaapveld et al., 1995 [42] (23)	6	ol	13, 10	Response	OR = 0.79 (0.35, 1.75)	Non-significant difference	High
		Harris et al., 1991 [30] (69)	6	db	35, 34				
		Kostiukova et al., 2003 [43] (60)	6	ol	30, 30				
		Remick et al., 1994 [39] (33)	7	db	16, 17				
Omori et al., 2010 [40]	MA	Barge-Schaapveld et al., 1995 [42] (23)	6	ol	13, 10	Remission	OR = 0.61 (0.28, 1.31)	Non-significant difference	High
		Harris et al., 1991 [30] (69)	6	db	35, 34				
		Kostiukova et al., 2003 [43] (60)	6	ol	30, 30				
		Remick et al., 1994 [39] (33)	7	db	16, 17				
Omori et al., 2009 [46]	MA	Barge-Schaapveld et al., 1995 [42] (23)	6	ol	13, 10	Response	RR = 0.91 (0.61, 1.38)	Non-significant difference	High
		Harris et al., 1991 [30] (69)	6	db	35, 34				
		Kostiukova et al., 2003 [43] (60)	6	Ol	30, 30				
		Remick et al., 1994 [39] (33)	7	db	16, 17				
Omori et al., 2009 [46]	MA	Barge-Schaapveld et al., 1995 [42] (23)	6	Ol	13, 10	Remission	RR = 0.74 (0.42, 1.30)	Non-significant difference	High
		Harris et al., 1991 [30] (69)	6	db	35, 34				

		Kostiukova et al., 2003 [43] (60)	6	Ol	30, 30				
		Remick et al., 1994 [39] (33)	7	db	16, 17				
Cipriani et al., 2018 [13]	NMA	Harris et al., 1991 [30] (69)	6	db	35, 34	Response	OR = 1.25 (0.99, 1.59)	Non-significant difference	High
		Murasaki et al., 1998 [55] (235)	4	db	113, 122				
		Remick et al., 1994 [39] (33)	7	db	16, 17				
Cipriani et al., 2018 [13]	NMA	Harris et al., 1991 [30] (69)	6	db	35, 34	Remission	OR = 1.07 (0.61, 1.88)	Non-significant difference	High
		Murasaki et al., 1998 [55] (235)	4	db	113, 122				
		Remick et al., 1994 [39] (33)	7	db	16, 17				
**Nortriptyline**<br>*Primary studies overlap = 100% (very high overlap)*									
Omori et al., 2010 [40]	MA	Otsubo et al., 2005 [44] (74)	8	Ol	36, 38	Response	OR = 0.91 (0.36, 2.28)	Non-significant difference	High
Omori et al., 2010 [40]	MA	Otsubo et al., 2005 [44] (74)	8	Ol	36, 38	Remission	OR = 1.78 (0.67, 4.77)	Non-significant difference	High
Omori et al., 2009 [46]	MA	Otsubo et al., 2005 [44] (74)	8	Ol	36, 38	Response	RR = 0.96 (0.57, 1.62)	Non-significant difference	High
Omori et al., 2009 [46]	MA	Otsubo et al., 2005 [44] (74)	8	Ol	36, 38	Remission	RR = 1.48 (0.61, 3.57)	Non-significant difference	High
**Imipramine, Desipramine or Dothiepin**									
Anderson and Tomenson , 1994 [5]	MA	Amin et al., 1984 [6] (314)	4-6	db	161, 153	Reduction in HAMD score	ES = 0.03 (-0.11, 0.17)	Non-significant difference	Low
		Amore et al., 1989 [23] (30)	4	db	15, 15				
		Feighner et al., 1989 [7] (48)	6	db	21, 27				
		Gonella et al., 1990 [27] (20)	4	db	10, 10				
		Guelfi et al., 1983 [28] (127)	4	db	59, 68				
		Guy et al., 1991 [29] (18)	4-6	db	10, 8				
		Lydiard et al., 1989 [8] (36)	6	db	18, 18				
		March et al., 1990 [9] (36)	6	db	18, 18				
		Mullin et al., 1988 [32] (50)	6	db	26, 24				

		Nathan et al., 1990 [33] (40)	4	db	20, 20				
		Rahman et al., 1991 [34] (36)	6	db	17, 19				
		Roth et al., 1990 [10] (51)	6	db	27, 24				
**Dothiepin**<br>*Primary studies overlap = 100% (very high overlap)*									
Omori et al., 2009 [46]	MA	Mullin et al., 1988 [32] (73)	6	db	37, 36	Response	RR = 1.05 (0.65, 1.69)	Non-significant difference	High
		Rahman et al., 1991 [34] (52)	6	db	26, 26				
Omori et al., 2009 [46]	MA	Mullin et al., 1988 [32] (73)	6	db	37, 36	Remission	RR = 1.05 (0.48, 2.25)	Non-significant difference	High
		Rahman et al., 1991 [34] (52)	6	db	26, 26				
Omori et al., 2010 [40]	MA	Mullin et al., 1988 [32] (73)	6	db	37, 36	Response	OR = 1.11 (0.55, 2.24)	Non-significant difference	High
		Rahman et al., 1991 [34] (52)	6	db	26, 26				
Omori et al., 2010 [40]	MA	Mullin et al., 1988 [32] (73)	6	db	37, 36	Remission	OR = 1.06 (0.48, 2.35)	Non-significant difference	High
		Rahman et al., 1991 [34] (52)	6	db	26, 26				
**Desipramine**<br>*Primary studies overlap = 100% (very high overlap)*									
Omori et al., 2010 [40]	MA	Tourigny-Rivard et al., 1996 [45] (47)	10	db	22, 25	Response	OR = 4.22 (0.98, 18.13)	Non-significant difference	High
Omori et al., 2010 [40]	MA	Tourigny-Rivard et al., 1996 [45] (47)	10	db	22, 25	Remission	OR = 4.5 (1.31,15.42)	Fluvoxamine superior to desipramine	High
Omori et al., 2009 [46]	MA	Tourigny-Rivard et al., 1996 [45] (47)	10	db	22,25	Response	RR = 1.44 (0.90, 2.31)	Non-significant difference	High
Omori et al., 2009 [46]	MA	Tourigny-Rivard et al., 1996 [45] (47)	10	db	22,25	Remission	RR = 2.27 (0.90, 5.73)	Non-significant difference	High
**Fluvoxamine *vs* SSRIs**									
**Unspecified SSRIs**									
	MA	Ansseau et al., 1994 [56] (120)	6	db	64, 56	Response	OR = 0.96 (0.74, 1.25)	Non-significant difference	High

Omori et al., 2010 [40]		Kato et al., 2006 [57] (101)	6	ol	49, 52				
		Kiev & Feiger, 1997 [58] (60)	7	db	30, 30				
		Nemeroff et al., 1995 [59] (97)	7	db	49,48				
		Rossini et al., 2005 [60] (88)	7	db	40, 48				
		Dalery & Honig, 2003 [61] (184)	6	db	90, 94				
		Rapaport et al., 1996 [62] (100)	7	db	51, 49				
		Haffmans et al., 1996 [63] (217)	6	db	109,108				
Omori et al., 2010 [40]	MA	Ansseau et al., 1994 [56] (120)	6	db	64, 56	Remission	OR = 0.98 (0.71, 1.37)	Non-significant difference	High
		Kato et al., 2006 [57] (101)	6	ol	49, 52				
		Kiev & Feiger, 1997 [58] (60)	7	db	30, 30				
		Nemeroff et al., 1995 [59] (97)	7	db	49,48				
		Rossini et al., 2005 [60] (88)	7	db	40, 48				
		Dalery & Honig, 2003 [61] (184)	6	db	90, 94				
		Rapaport et al., 1996 [62] (100)	7	db	51, 49				
		Haffmans et al., 1996 [63] (217)	6	db	109,108				
**Paroxetine** *Primary studies overlap = 89% (very high overlap)*									
Omori et al., 2010 [40]	MA	Ansseau et al., 1994 [56] (120)	6	db	64, 56	Response	OR = 0.83 (0.51, 1.34)	Non-significant difference	High
		Kato et al., 2006 [57] (101)	6	ol	49, 52				
		Kiev & Feiger, 1997 [58] (60)	7	db	30, 30				
Omori et al., 2010 [40]	MA	Ansseau et al., 1994 [56] (120)	6	db	64, 56	Remission	OR = 0.77 (0.45, 1.33)	Non-significant difference	High
		Kato et al., 2006 [57] (101)	6	ol	49, 52				
		Kiev & Feiger, 1997 [58] (60)	7	db	30, 30				

Omori et al., 2009 [46]	MA	Ansseau et al., 1994 [56] (120)	6	db	64, 56	Response	RR = 0.92 (0.70, 1.21)	Non-significant difference	High
		Kiev & Feiger, 1997 [58] (60)	7	db	30, 30				
		Kato et al., 2006 [57] (101)	6	ol	49, 52				
Omori et al., 2009 [46]	MA	Ansseau et al., 1994 [56] (120)	6	db	64, 56	Remission	RR = 0.83 (0.52, 1.31)	Non-significant difference	High
		Kiev & Feiger, 1997 [58] (60)	7	db	30, 30				
		Kato et al., 2006 [57] (101)	6	ol	49, 52				
Cipriani et al., 2009 [64]	MA	Ansseau et al., 1994 [56] (120)	6	db	64, 56	Response	OR = 0.83 (0.51, 1.34)	Non-significant difference	High
		Kato et al., 2006 [57] (80)	6	db	41, 39				
		Kiev & Feiger, 1997 [58] (60)	7	db	30, 30				
Cipriani et al., 2018 [13]	NMA	Ansseau et al., 1994 [56] (120)	6	db	64, 56	Response	OR = 0.84 (0.67, 1.04)	Non-significant difference	High
		Kiev & Feiger, 1997 [58] (60)	7	db	30, 30				
Cipriani et al., 2018 [13]	NMA	Ansseau et al., 1994 [56] (120)	6	db	64, 56	Remission	OR = 1.13 (0.50, 2.46)	Non-significant difference	High
		Kiev & Feiger, 1997 [58] (60)	7	db	30, 30				
**Sertraline**<br>*Primary studies overlap = 88% (very high overlap)*									
Omori et al., 2010 [40]	MA	Nemeroff et al., 1995 [59] (97)	7	db	49,48	Response	OR = 1.21 (0.53, 2.75)	Non-significant difference	High
		Rossini et al., 2005 [60] (88)	7	db	40, 48				
Omori et al., 2010 [40]	MA	Nemeroff et al., 1995 [59] (97)	7	db	49,48	Remission	OR = 1.31 (0.48, 3.57)	Non-significant difference	High
		Rossini et al., 2005 [60] (88)	7	db	40, 48				
Omori et al., 2009 [46]	MA	Nemeroff et al., 1995 [59] (97)	7	db	49,48	Response	RR = 1.10 (0.71, 1.70)	Non-significant difference	High
		Rossini et al., 2005 [60] (88)	7	db	40, 48				
Omori et al., 2009 [46]	MA	Nemeroff et al., 1995 [59] (97)	7	db	49,48	Remission	RR = 1.16 (0.63, 2.15)	Non-significant difference	High
		Rossini et al., 2005 [60] (88)	7	db	40, 48				
Ramsberg et al., 2012 [48]	NMA	Rossini et al., 2005 [60] (88)	7	db	40, 48	Probability of remission	OR = 1.41 (0.92, 2.10)	Non-significant difference	High

Cipriani et al., 2009 [64]	NMA	Nemeroff et al., 1995 [59] (97)	7	db	49,48	Response	OR = 1.21 (0.53, 2.75)	Sertraline significantly more effective than fluvoxamine	High
		Rossini et al., 2005 [60] (88)	7	db	40, 48				
Cipriani et al., 2018 [13]	NMA	Nemeroff et al., 1995 [59] (97)	7	db	49,48	Response	OR = 0.89 (0.70, 1.13)	Non-significant difference	High
		Rossini et al., 2005 [60] (88)	7	db	40, 48				
Cipriani et al., 2018 [13]	NMA	Nemeroff et al., 1995 [59] (97)	7	db	49,48	Remission	OR = 0.68 (0.34, 1.36)	Non-significant difference	High
		Rossini et al., 2005 [60] (88)	7	db	40, 48				
**Fluoxetine**<br>*Primary studies overlap = 100% (very high overlap)*									
Omori et al., 2009 [46]	MA	Dalery & Honig, 2003 [61] (184)	6	db	90, 94	Response	RR = 1.00 (0.78, 1.28)	Non-significant difference	High
		Rapaport et al., 1996 [62] (100)	7	db	51, 49				
Omori et al., 2009 [46]	MA	Dalery & Honig, 2003 [61] (184)	6	db	90, 94	Remission	RR = 1.15 (0.72, 1.82)	Non-significant difference	High
		Rapaport et al., 1996 [62] (100)	7	db	51, 49				
Omori et al., 2010 [40]	MA	Dalery & Honig, 2003 [61] (184)	6	db	90, 94	Response	OR = 1.00 (0.62, 1.61)	Non-significant difference	High
		Rapaport et al., 1996 [62] (100)	7	db	51, 49				
Omori et al., 2010 [40]	MA	Dalery & Honig, 2003 [61] (184)	6	db	90, 94	Remission	OR = 1.24 (0.74, 2.06)	Non-significant difference	High
		Rapaport et al., 1996 [62] (100)	7	db	51, 49				
Cipriani et al., 2009 [64]	MA	Rapaport et al., 1996 [62] (100)	7	db	51, 49	Response	OR = 1.03 (0.64, 1.66)	Non-significant difference	High
		Dalery & Honig, 2003 [61] (184)	6	db	90, 94				
Cipriani et al., 2018 [13]	NMA	Dalery & Honig, 2003 [61] (184)	6	db	90, 94	Response	OR= 1.00 (0.80, 1.25)	Non-significant difference	High
		Rapaport et al., 1996 [62] (100)	7	db	51, 49				

Cipriani et al., 2018 [13]	NMA	Dalery & Honig, 2003 [61] (184)	6	db	90, 94	Remission	OR= 0.85 (0.47, 1.51)	Non-significant difference	High
		Rapaport et al., 1996 [62] (100)	7	db	51, 49				
**Citalopram**<br>*Primary studies overlap = 100% (very high overlap)*									
Omori et al., 2010 [40]	MA	Haffmans et al., 1996 [63] (217)	6	db	109,108	Response	OR = 0.90 (0.50, 1.62)	Non-significant difference	High
Omori et al., 2010 [40]	MA	Haffmans et al., 1996 [63] (217)	6	db	109,108	Remission	OR = 0.56 (0.23, 1.34)	Non-significant difference	High
Omori et al., 2009 [46]	MA	Haffmans et al., 1996 [63] (217)	6	db	109,108	Response	RR = 0.93 (0.54, 1.60)	Non-significant difference	High
Omori et al., 2009 [46]	MA	Haffmans et al., 1996 [63] (217)	6	db	109,108	Remission	RR = 0.59 (0.21, 1.66)	Non-significant difference	High
Ramsberg et al., 2012 [48]	NMA	Haffmans et al., 1996 [63] (217)	6	db	109, 108	Probability of remission	OR = 0.802 (0.511, 1.195)	Non-significant difference	High
Cipriani et al., 2009 [64]	MA	Haffmans et al., 1996 [63] (217)	6	db	109, 108	Response	OR = 0.90 (0.50, 1.62)	Non-significant difference	High
Cipriani et al., 2018 [13]	NMA	Haffmans et al., 1996 [63] (217)	6	db	109, 108	Response	OR = 1.06 (0.82, 1.39)	Non-significant difference	High
Cipriani et al., 2018 [13]	NMA	Haffmans et al., 1996 [63] (217)	6	db	109, 108	Remission	OR = 1.84 (0.72, 5.00)	Non-significant difference	High
**Fluvoxamine *vs* SNRIs**									
**Unspecified SNRIs**<br>*Primary studies overlap = 50% (very high overlap)*									

Omori et al., 2010 [40]	MA	Clerc & the Milnacipran/ Fluvoxamine Study group, 2001 [65] (113)	6	db	56, 57	Response	OR = 0.48 (0.27,0.85)	SNRIs superior to fluvoxamine	High
		Hacket et al., 1998 [66]* (71)	6	db	34, 37				
		Hacket et al., 1998 [66]* (74)	6	db	34, 40				
Omori et al., 2010 [40]	MA	Clerc & the Milnacipran/ Fluvoxamine Study group, 2001 [65] (113)	6	db	56, 57	Remission	OR = 0.61 (0.34, 1.08)	Non-significant difference	High
		Hacket et al., 1998 [66]* (71)	6	db	34, 37				
		Hacket et al., 1998 [66]* (74)	6	db	34, 40				
Omori et al., 2009 [46]	MA	Clerc & the Milnacipran/ Fluvoxamine Study group, 2001 [65] (113)	6	db	56, 57	Response	RR = 0.76 (0.56, 1.04)	Non-significant difference	High
		Hackett et al., 1998 [66] (111)	6	db	34, 77				
Omori et al., 2009 [46]	MA	Clerc & the Milnacipran/ Fluvoxamine Study group, 2001 [65] (113)	6	db	56, 57	Remission	RR = 0.73 (0.45, 1.20)	Non-significant difference	High
		Hackett et al., 1998 [66] (111)	6	db	34, 77				
**Milnacipram**<br>*Primary studies overlap = 50% (very high overlap)*									
Omori et al., 2010 [40]	MA	Clerc & the Milnacipran/ Fluvoxamine Study group, 2001 [65] (113)	6	db	56, 57	Response	OR = 0.57 (0.26, 1.23)	Non-significant difference	High
Omori et al., 2010 [40]	MA	Clerc & the Milnacipran/ Fluvoxamine Study group, 2001 [65] (113)	6	db	56, 57	Remission	OR = 0.68 (0.3,1.51)	Non-significant difference	High
Omori et al., 2009 [46]	MA	Clerc & the Milnacipran/ Fluvoxamine Study group, 2001 [65] (113)	6	db	56, 57	Response	RR = 0.81 (0.56, 1.18)	Non-significant difference	High
Omori et al., 2009 [46]	MA	Clerc & the Milnacipran/ Fluvoxamine Study group, 2001 [65] (113)	6	db	56, 57	Remission	RR = 0.76 (0.37, 1.59)	Non-significant difference	High
Nakagawa et al., 2009 [67]	MA	Clerc & the Milnacipran/ Fluvoxamine Study group, 2001 [65] (113)	6	db	56, 57	Response	OR = 1.76 (0.81, 3.83)	Non-significant difference	High

Nakagawa et al., 2009 [67]	MA	Clerc & the Milnacipran/ Fluvoxamine Study group, 2001 [65] (113)	6	db	56, 57	Remission	OR = 1.48 (0.66, 3.3)	Non-significant difference	High
Lopez-Ibor et al., 1996 [68]	MA	Unpublished clinical trial (113) / Clerc & the Milnacipran/ Fluvoxamine Study group, 2001 [65] (113)	6	db	56,57	Response	HAMD score (*P* = 0.09)<br>MADRS score (*P* = 0.01)	Milnacipram significantly more effective than fluvoxamine	Low
Lopez-Ibor et al., 1996 [68]	MA	Unpublished clinical trial (113) / Clerc & the Milnacipran/ Fluvoxamine Study group, 2001 [65] (113)	6	db	56,57	Remission	Milnacipram = 47%<br>Fluvoxamine = 36%<br>P = 0.20	Non-significant difference	Low
Cipriani et al., 2009 [64]	MA	Clerc & the Milnacipran/ Fluvoxamine Study group, 2001 [65] (113)	6	db	56, 57	Response	OR = 0.57 (0.26, 1.23)	Non-significant difference	High
Cipriani et al., 2018 [13]	NMA	Ansseau et al., 1991 [69] (126)	4	db	41, 85	Response	OR = 0.89 (0.67, 1.17)	Non-significant difference	High
		Clerc & the Milnacipran/ Fluvoxamine Study group, 2001 [65] (113)	6	db	56, 57				
**Venlafaxine**<br>*Primary studies overlap = 33% (very high overlap)*									
Cipriani et al., 2009 [64]	MA	Hackett et al., 1998 [66] (111)	6	db	34, 77	Response	OR = 0.42 (0.19, 0.96)	Venlafaxine significantly more effective than fluvoxamine	High
Omori et al., 2010 [40]	MA	Hackett et al., 1998 [66]* (71)	6	db	34, 37	Response	OR = 0.40 (0.18, 0.92)	Venlafaxine significantly more effective than fluvoxamine	High
		Hackett et al., 1998 [66]* (74)	6	db	34, 40				
Omori et al., 2010 [40]	MA	Hackett et al., 1998 [66]* (71)	6	db	34, 37	Remission	OR = 0.54 (0.23, 1.24)	Non-significant difference	High
		Hackett et al., 1998 [66]* (74)	6	db	34, 40				
Omori et al., 2009 [46]	MA	Hackett et al., 1998 [66] (111)	6	db	34, 77	Response	RR = 0.65 (0.37, 1.15)	Non-significant difference	High

Omori et al., 2009 [46]	MA	Hackett et al., 1998 [66] (111)	6	db	34, 77	Remission	RR = 0.70 (0.36, 1.37)	Non-significant difference	High
Nemeroff et al., 2008 [70]	MA	Hackett et al., 1998 [66] (111)	6	db	34, 77	Remission (HAMD total score of 7 or less)	RD = 0.141 (-0.055, 0.336)	Non-significant difference	Moderate
Ramsberg et al., 2012 [48]	NMA	Hackett et al., 1998 [66] (111)	6	db	34, 77	Probability of remission	OR = 1.623 (1.062, 2.387)	Venlafaxine significantly more effective than fluvoxamine	High
Cipriani et al., 2018 [13]	NMA	Unpublished clinical trial - 0600A-347	NK	NK	NK	Response	OR = 0.84 (0.66, 1.07)	Non-significant difference	High
		Hackett et al., 1998 [66] (111)	6	db	34, 77				
Cipriani et al., 2018 [13]	NMA	Unpublished clinical trial - 0600A-347	NK	NK	NK	Remission	OR = 1.93 (0.76, 5.01)	Non-significant difference	High
		Hackett et al., 1998 [66] (111)	6	db	34, 77				
**Fluvoxamine *vs* Other Antidepressants**									
**Mirtazapine**<br>*Primary studies overlap = 22% (very high overlap)*									
Omori et al., 2009 [46]	MA	Schoemaker et al., 2002 [71] (412)	6	db	207, 205	Response	RR = 0.95 (0.78,1.16)	Non-significant difference	High
Omori et al., 2009 [46]	MA	Schoemaker et al., 2002 [71] (412)	6	db	207, 205	Remission	RR = 1.10 (0.83, 1.45)	Non-significant difference	High
Omori et al., 2010 [40]	MA	Schoemaker et al., 2002 [71] (412)	6	db	207, 205	Response	OR = 0.72 (0.47, 1.11)	Non-significant difference	High
Omori et al., 2010 [40]	MA	Schoemaker et al., 2002 [71] (412)	6	db	207, 205	Remission	OR = 1.19 (0.81, 1.76)	Non-significant difference	High
Cipriani et al., 2009 [64]	NMA	Schoemaker et al., 2002 [71] (412)	6	db	207, 205	Response	OR = 0.88 (0.59, 1.31)	Mirtazapine significantly more effective than fluvoxamine	High
	NMA	Murasaki et al., 2010a [72] (209)	6	db	105, 104	Response	OR= 0.78 (0.60, 0.99)		High

Cipriani et al., 2018 [13]		Murasaki et al., 2010b [72] (203)	6	db	102, 101			Mirtazapine significantly more effective than fluvoxamine	
Cipriani et al., 2018 [13]	NMA	Murasaki et al., 2010a [72] (209)	6	db	105, 104	Remission	OR = 0.84 (0.52, 1.36)	Non-significant difference	High
		Murasaki et al., 2010b [72] (203)	6	db	102, 101				
**Mianserin** *Primary studies overlap = 100% (very high overlap)*									
Omori et al., 2009 [46]	MA	Moon & Jesinger, 1991 [73] (62)	6	db	31, 31	Response	RR = 1.09 (0.86, 1.40)	Non-significant difference	High
		Perez & Ashford, 1990 [74] (63)	6	db	30, 33				
Omori et al., 2009 [46]	MA	Moon & Jesinger, 1991 [73] (62)	6	db	31, 31	Remission	RR = 1.16 (0.93, 1.44)	Non-significant difference	High
		Perez & Ashford, 1990 [74] (63)	6	db	30, 33				
Omori et al. 2010 [40]	MA	Moon & Jesinger, 1991 [73] (62)	6	db	31, 31	Response	OR = 1.25 (0.55, 2.87)	Non-significant difference	High
		Perez & Ashford, 1990 [74] (63)	6	db	30, 33				
Omori et al., 2010 [40]	MA	Moon & Jesinger, 1991 [73] (62)	6	db	31, 31	Remission	OR = 2.02 (0.55, 7.39)	Non-significant difference	High
		Perez & Ashford, 1990 [74] (63)	6	db	30, 33				
**Moclobemide**									
Suchting et al., 2021 [75]	NMA	2 RCTs were identified, but no specific information was obtained	NK	db	NK	Response	OR = 0.53 (0.21, 1.33)	Non-significant difference	Moderate

[1] B.J. Shea, B.C. Reeves, G. Wells, M. Thuku, C. Hamel, J. Moran, D. Moher, P. Tugwell, V. Welch, E. Kristjansson, D.A. Henry, AMSTAR 2: a critical appraisal tool for systematic reviews that include randomised or non-randomised studies of healthcare interventions, or both, BMJ 358 (2017) j4008.

[2] Z. Yuan, Z. Chen, M. Xue, J. Zhang, L. Leng, Application of antidepressants in depression: A systematic review and meta-analysis, J Clin Neurosci 80 (2020) 169-181.

[3] J.I. Hudson, S.L. McElroy, N.C. Raymond, S. Crow, P.E. Keck, W.P. Carter, J.E. Mitchell, S.M. Strakowski, H.G. Pope, B.S. Coleman, J.M. Jonas, Fluvoxamine in the treatment of binge-eating disorder: a multicenter placebo-controlled, double-blind trial, Am J Psychiatry 155(12) (1998) 1756-62.

[4] D.J. Stein, H.G. Westenberg, H. Yang, D. Li, L.M. Barbato, Fluvoxamine CR in the long-term treatment of social anxiety disorder: the 12- to 24-week extension phase of a multicentre, randomized, placebo-controlled trial, Int J Neuropsychopharmacol 6(4) (2003) 317-23.

[5] I.M. Anderson, B.M. Tomenson, The efficacy of selective serotonin re-uptake inhibitors in depression: a meta-analysis of studies against tricyclic antidepressants, J Psychopharmacol 8(4) (1994) 238-49.

[6] Amin M M, Ananth J V, Coleman B S, Darcourt G, Farkas T, Goldstein B, Lapierre Y D, Paykel E, Wakelin J S. Fluvoxamine: antidepressant effects confirmed in a placebo-controlled international study. Clin Neuropharmacol 1984; 7(Suppl 1): S316-S317.

[7] J.P. Feighner, W.F. Boyer, C.H. Meredith, G.G. Hendrickson, A placebo-controlled inpatient comparison of fluvoxamine maleate and imipramine in major depression, Int Clin Psychopharmacol 4(3) (1989) 239-44.

[8] R.B. Lydiard, L.K. Laird, W.A. Morton, T.E. Steele, C. Kellner, M.T. Laraia, J.C. Ballenger, Fluvoxamine, imipramine, and placebo in the treatment of depressed outpatients: effects on depression, Psychopharmacol Bull 25(1) (1989) 68-70.

[9] J.S. March, K.A. Kobak, J.W. Jefferson, J. Mazza, J.H. Greist, A double-blind, placebo-controlled trial of fluvoxamine versus imipramine in outpatients with major depression, J Clin Psychiatry 51(5) (1990) 200-2.

[10] D. Roth, J. Mattes, K.H. Sheehan, D.V. Sheehan, A double-blind comparison of fluvoxamine, desipramine and placebo in outpatients with depression, Prog Neuropsychopharmacol Biol Psychiatry 14(6) (1990) 929-39.

[11] T. Kishi, T. Ikuta, K. Sakuma, M. Okuya, M. Hatano, Y. Matsuda, N. Iwata, Antidepressants for the treatment of adults with major depressive disorder in the maintenance phase: a systematic review and network meta-analysis, Mol Psychiatry 28(1) (2023) 402-409.

[12] J.L. Terra, S.A. Montgomery, Fluvoxamine prevents recurrence of depression: results of a long-term, double-blind, placebo-controlled study, International clinical psychopharmacology 13(2) (1998) 55-62.

[13] A. Cipriani, T.A. Furukawa, G. Salanti, A. Chaimani, L.Z. Atkinson, Y. Ogawa, S. Leucht, H.G. Ruhe, E.H. Turner, J.P.T. Higgins, M. Egger, N. Takeshima, Y. Hayasaka, H. Imai, K. Shinohara, A. Tajika, J.P.A. Ioannidis, J.R. Geddes, Comparative Efficacy and Acceptability of 21 Antidepressant Drugs for the Acute Treatment of Adults With Major Depressive Disorder: A Systematic Review and Network Meta-Analysis, Focus (Am Psychiatr Publ) 16(4) (2018) 420-429.

[14] W.A. Brown, M. Arato, R. Shrivastava, Pituitary-adrenocortical hyperfunction and intolerance to fluvoxamine, a selective serotonin uptake inhibitor, Am J Psychiatry 143(1) (1986) 88-90.

[15] G.B. Cassano, L. Conti, G. Massimetti, F. Mengali, J.S. Waekelin, J. Levine, Use of a standardized documentation system (BLIPS/BDP) in the conduct of a multicenter international trial comparing fluvoxamine, imipramine, and placebo, Psychopharmacol Bull 22(1) (1986) 52-8.

[16] J.L. Claghorn, C.Q. Earl, D.D. Walczak, K.A. Stoner, L.F. Wong, D. Kanter, V.P. Houser, Fluvoxamine maleate in the treatment of depression: a single-center, double-blind, placebo-controlled comparison with imipramine in outpatients, J Clin Psychopharmacol 16(2) (1996) 113-20.

[17] R.A. Dominguez, B.J. Goldstein, A.F. Jacobson, R.M. Steinbook, A double-blind placebo-controlled study of fluvoxamine and imipramine in depression, J Clin Psychiatry 46(3) (1985) 84-7.

[18] L. Fabre, L.J. Birkhimer, B.A. Zaborny, L.F. Wong, B.M. Kapik, Fluvoxamine versus imipramine and placebo: a double-blind comparison in depressed patients, Int Clin Psychopharmacol 11(2) (1996) 119-27.

[19] T.M. Itil, R.K. Shrivastava, S. Mukherjee, B.S. Coleman, S.T. Michael, A double-blind placebo-controlled study of fluvoxamine and imipramine in out-patients with primary depression, Br J Clin Pharmacol 15 Suppl 3(Suppl 3) (1983) 433S-438S.

[20] Y.D. Lapierre, M. Browne, E. Horn, L.K. Oyewumi, D. Sarantidis, N. Roberts, K. Badoe, P. Tessier, Treatment of major affective disorder with fluvoxamine, J Clin Psychiatry 48(2) (1987) 65-8.

[21] K.R. Norton, L.I. Sireling, A.V. Bhat, B. Rao, E.S. Paykel, A double-blind comparison of fluvoxamine, imipramine and placebo in depressed patients, J Affect Disord 7(3-4) (1984) 297-308.

[22] D.D. Walczak, J.T. Apter, J.A. Halikas, R.L. Borison, J.S. Carman, G.L. Post, R. Patrick, J.B. Cohn, L.A. Cunningham, B. Rittberg, S.H. Preskorn, J.S. Kang, C.S. Wilcox, The oral dose-

effect relationship for fluvoxamine: a fixed-dose comparison against placebo in depressed outpatients, Ann Clin Psychiatry 8(3) (1996) 139-51.

[23] Amore M, Bellini M, Berardi D, Berlinzani L, Cervino

G, Cremonini A, Ferrari G, Innamorati A. Double-blind comparison of fluvoxamine

and imipramine in depressed patients. Curr Ther Res 1989; 4:6 815-820.

[24] J.E. De Wilde, D.P. Doogan, Fluvoxamine and chlorimipramine in endogenous depression, J Affect Disord 4(3) (1982) 249-59.

[25] J.E. De Wilde, C. Mertens, J.S. Wakelin, Clinical trials of fluvoxamine vs chlorimipramine with single and three times daily dosing, Br J Clin Pharmacol 15 Suppl 3(Suppl 3) (1983) 427S-431S.

[26] P. Dick, E. Ferrero, A double-blind comparative study of the clinical efficacy of fluvoxamine and chlorimipramine, Br J Clin Pharmacol 15 Suppl 3(Suppl 3) (1983) 419S-425S.

[27] G. Gonella, G. Baignoli, U. Ecari, Fluvoxamine and imipramine in the treatment of depressive patients: a double-blind controlled study, Curr Med Res Opin 12(3) (1990) 177-84.

[28] J.D. Guelfi, J.F. Dreyfus, P. Pichot, A double-blind controlled clinical trial comparing fluvoxamine with imipramine, Br J Clin Pharmacol 15 Suppl 3(Suppl 3) (1983) 411S-417S.

[29] W. Guy, W.H. Wilson, T.A. Ban, D.L. King, G. Manov, O.K. Fjetland, A double-blind clinical trial of fluvoxamine and imipramine in patients with primary depression, Psychopharmacol Bull 20(1) (1984) 73-8.

[30] Harris B, Szulecka T K, Anstee J A. Fluvoxamine versus amitriptyline in depressed hospital out-patients: A multicentre double-blind comparative trial. Br J Clin Res 1991; 2: 89-99.

[31] C.J. Klok, G.J. Brouwer, H.M. van Praag, D. Doogan, Fluvoxamine and clomipramine in depressed patients. A double-blind clinical study, Acta Psychiatr Scand 64(1) (1981) 1-11.

[32] J.M. Mullin, V.R. Pandita-Gunawardena, A.M. Whitehead, A double-blind comparison of fluvoxamine and dothiepin in the treatment of major affective disorder, Br J Clin Pract 42(2) (1988) 51-5.

[33] R.S. Nathan, J.M. Perel, B.G. Pollock, D.J. Kupfer, The role of neuropharmacologic selectivity in antidepressant action: fluvoxamine versus desipramine, J Clin Psychiatry 51(9) (1990) 367-72.

[34] M.K. Rahman, M.J. Akhtar, N.C. Savla, R.R. Sharma, J.M. Kellett, J.J. Ashford, A double-blind, randomised comparison of fluvoxamine with dothiepin in the treatment of depression in elderly patients, Br J Clin Pract 45(4) (1991) 255-8.

[35] I.M. Anderson, Selective serotonin reuptake inhibitors versus tricyclic antidepressants: a meta-analysis of efficacy and tolerability, J Affect Disord 58(1) (2000) 19-36.

[36] M. Gasperini, F. Gatti, L. Bellini, R. Anniverno, E. Smeraldi, Perspectives in clinical psychopharmacology of amitriptyline and fluvoxamine. A double-blind study in depressed inpatients, Neuropsychobiology 26(4) (1992) 186-92.

[37] S. Kasper, G. Voll, A. Vieira, H. Kick, Response to total sleep deprivation before and during treatment with fluvoxamine or maprotiline in patients with major depression--results of a double-blind study, Pharmacopsychiatry 23(3) (1990) 135-42.

[38] E.A. Ottevanger, Fluvoxamine and clomipramine in depressed hospitalised patients: results from a randomised, double-blind study, Encephale 21(4) (1995) 317-21.

[39] R.A. Remick, R. Reesal, M. Oakander, J. Allen, J. Claman, C.E. Ramirez, K. Perry, F.D. Keller, Comparison of fluvoxamine and amitriptyline in depressed outpatients, Current Therapeutic Research 55(3) (1994) 243-250.

[40] I.M. Omori, N. Watanabe, A. Nakagawa, A. Cipriani, C. Barbui, H. McGuire, R. Churchill, T.A. Furukawa, Fluvoxamine versus other anti-depressive agents for depression, Cochrane Database Syst Rev (3) (2010) CD006114.

[41] J. Zohar, H. Keegstra, L. Barrelet, Fluvoxamine as effective as clomipramine against symptoms of severe depression: results from a multicentre, double-blind study, Hum Psychopharmacol 18(2) (2003) 113-9.

[42] D.Q. Barge-Schaapveld, N.A. Nicolson, R.G. van der Hoop, M.W. De Vries, Changes in daily life experience associated with clinical improvement in depression, J Affect Disord 34(2) (1995) 139-54.

[43] E.G. Kostiukova, G.M. Granenov, L.A. Andreĭchik, O.V. Serditov, S.N. Mosolov, [Comparative efficacy and tolerance of fluvoxamine and amitriptyline in the treatment of moderate and severe depression in mental hospital], Zh Nevrol Psikhiatr Im S S Korsakova 103(1) (2003) 24-9.

[44] T. Otsubo, Y. Akimoto, H. Yamada, R. Koda, H. Aoyama, K. Tanaka, M. Mimura, K. Nakagome, K. Kamijima, A comparative study of the efficacy and safety profiles between fluvoxamine and nortriptyline in Japanese patients with major depression, Pharmacopsychiatry 38(1) (2005) 30-5.

[45] Tourigny-Rivard, M.; Nair, N.; Vincent, P. Fluvoxamine versus desipramine in elderly patients with major depression: A double-blind comparison. 9th ECNP (European College of Neuropsychopharmacology) Congress; Amsterdam, Netherlands. 1996.

[46] I.M. Omori, N. Watanabe, A. Nakagawa, T. Akechi, A. Cipriani, C. Barbui, H. McGuire, R. Churchill, T.A. Furukawa, M.-A.o.N.G.A.M.S. Group, Efficacy, tolerability and side-effect profile of fluvoxamine for major depression: meta-analysis, J Psychopharmacol 23(5) (2009) 539-50.

[47] H.L. Miller, R.D. Ekstrom, G.A. Mason, R.B. Lydiard, R.N. Golden, Noradrenergic function and clinical outcome in antidepressant pharmacotherapy, Neuropsychopharmacology 24(6) (2001) 617-23.

[48] J. Ramsberg, C. Asseburg, M. Henriksson, Effectiveness and cost-effectiveness of antidepressants in primary care: a multiple treatment comparison meta-analysis and cost-effectiveness model, PLoS One 7(8) (2012) e42003.

[49] T.K. Birkenhäger, W.W. van den Broek, P.G. Mulder, J.A. Bruijn, P. Moleman, Comparison of two-phase treatment with imipramine or fluvoxamine, both followed by lithium addition, in inpatients with major depressive disorder, Am J Psychiatry 161(11) (2004) 2060-5.

[50] H.J. Möller, J. Fuger, S. Kasper, Efficacy of new generation antidepressants: meta-analysis of imipramine-controlled studies, Pharmacopsychiatry 27(6) (1994) 215-23.

[51] Conti, L., G. F. Placidi, L. Dell 'Osso, A. Lenzi. G. B. Cassano. Therapeutic

response in subtypes of major depression. New Trends Exp. Clin. Psychiatry ,

1987; 101 - 107.

[52] Pöldinger, W, E. Bures. Fluvoxamine in patients with

depressive disorder. In: Proceedings of the international symposium on

fluvoxamine. Duphar Medical Publications, Bern 1984; p. 41 -44.

[53] Wagner, W, J Wakelin, B. S. Coleman, K. Cimander:

Therapeutische Ergebnisse mit Fluvoxamin und der Einfluß psychotroper

Begleitmedikation auf Wirksamkeit und Verträglichkeit. Adv. Pharrnacother.

(Basel) 2. 1985; 33-65.

[54] J.S. Wakelin, Fluvoxamine in the treatment of the older depressed patient; double-blind, placebo-controlled data, Int Clin Psychopharmacol 1(3) (1986) 221-30.

[55] Murasaki M, Mori A, Miura S et al. Clinical evaluation of SME3110 (fluvoxamine maleate) in the treatment of depression and depressive state: A double-blind, comparative study with amitriptyline. Rinsho-Iyaku (Journal of Clinical Therapeutics & Medicines) 1998; 14(5): 951-980.

[56] M. Ansseau, A. Gabriëls, J. Loyens, F. Bartholomé, J.L. Evrard, A. De Nayer, R. Linhart, J. Wirtz, F. Bruynooghe, K. Surinx, H. Clarysse, R. Marganne, P. Papart, Controlled comparison of paroxetine and fluvoxamine in major depression, Human Psychopharmacology: Clinical and Experimental 9(5) (1994) 329-336.

[57] M. Kato, T. Fukuda, M. Wakeno, K. Fukuda, G. Okugawa, Y. Ikenaga, M. Yamashita, Y. Takekita, K. Nobuhara, J. Azuma, T. Kinoshita, Effects of the serotonin type 2A, 3A and 3B receptor and the serotonin transporter genes on paroxetine and fluvoxamine efficacy and

adverse drug reactions in depressed Japanese patients, Neuropsychobiology 53(4) (2006) 186-95.

[58] A. Kiev, A. Feiger, A double-blind comparison of fluvoxamine and paroxetine in the treatment of depressed outpatients, J Clin Psychiatry 58(4) (1997) 146-52.

[59] C.B. Nemeroff, P.T. Ninan, J. Ballenger, R.B. Lydiard, J. Feighner, W.M. Patterson, J.H. Greist, Double-blind multicenter comparison of fluvoxamine versus sertraline in the treatment of depressed outpatients, Depression 3(4) (1995) 163-169.

[60] D. Rossini, A. Serretti, L. Franchini, L. Mandelli, E. Smeraldi, D. De Ronchi, R. Zanardi, Sertraline versus fluvoxamine in the treatment of elderly patients with major depression: a double-blind, randomized trial, J Clin Psychopharmacol 25(5) (2005) 471-5.

[61] J. Dalery, A. Honig, Fluvoxamine versus fluoxetine in major depressive episode: a double-blind randomised comparison, Hum Psychopharmacol 18(5) (2003) 379-84.

[62] M. Rapaport, E. Coccaro, Y. Sheline, T. Perse, P. Holland, L. Fabre, D. Bradford, A comparison of fluvoxamine and fluoxetine in the treatment of major depression, J Clin Psychopharmacol 16(5) (1996) 373-8.

[63] P.M. Haffmans, L. Timmerman, C.A. Hoogduin, Efficacy and tolerability of citalopram in comparison with fluvoxamine in depressed outpatients: a double-blind, multicentre study. The LUCIFER Group, Int Clin Psychopharmacol 11(3) (1996) 157-64.

[64] A. Cipriani, T.A. Furukawa, G. Salanti, J.R. Geddes, J.P. Higgins, R. Churchill, N. Watanabe, A. Nakagawa, I.M. Omori, H. McGuire, M. Tansella, C. Barbui, Comparative efficacy and acceptability of 12 new-generation antidepressants: a multiple-treatments meta-analysis, Lancet 373(9665) (2009) 746-58.

[65] G. Clerc, M.F.S. Group, Antidepressant efficacy and tolerability of milnacipran, a dual serotonin and noradrenaline reuptake inhibitor: a comparison with fluvoxamine, Int Clin Psychopharmacol 16(3) (2001) 145-51.

[66] Hackett D, Salinas E, Desmet A. Efficacy and safety of venlafaxine vs. fluvoxamine in outpatients with major depression. Eur Neuropsychopharmacol. 1998;8(Supp2):S209.

[67] A. Nakagawa, N. Watanabe, I.M. Omori, C. Barbui, A. Cipriani, H. McGuire, R. Churchill, T.A. Furukawa, Milnacipran versus other antidepressive agents for depression, Cochrane Database Syst Rev 2009(3) (2009) CD006529.

[68] J. Lopez-Ibor, J.D. Guelfi, Y. Pletan, A. Tournoux, J.F. Prost, Milnacipran and selective serotonin reuptake inhibitors in major depression, Int Clin Psychopharmacol 11 Suppl 4 (1996) 41-6.

[69] M. Ansseau, R. von Frenckell, M.-A. Gérard, C. Mertens, J. De Wilde, L. Botte, J.-M. Devoitille, J.-L. Evrard, A. De Nayer, P. Darimont, J. Mirel, B. Troisfontaines, C. Toussaint, J.-P. Couzinier, J.-P. Demarez, C. Serre, Interest of a loading dose of milnacipran in endogenous

depressive inpatients: Comparison with the standard regimen and with fluvoxamine, European Neuropsychopharmacology 1(2) (1991) 113-121.

[70] C.B. Nemeroff, R. Entsuah, I. Benattia, M. Demitrack, D.M. Sloan, M.E. Thase, Comprehensive analysis of remission (COMPARE) with venlafaxine versus SSRIs, Biol Psychiatry 63(4) (2008) 424-34.

[71] Schoemaker J, Gailledreau J, Hoyberg OJ. First, randomized, double-blind comparison of mirtazapine (15–45 mg) and fl uvoxamine (50–150 mg) in the treatment of depression. Int J Neuropsychopharmacol 2002; 5 (suppl 1): 140.

[72] Murasaki M, Schoemaker JH, Miyake K, Gailledreau J, Heukels AJ, Fennema HP, Sitsen JMA. Comparison of efficacy and safety of mirtazapine versus fluvoxamine in Japanese and Caucasian patients with major depressive disorder. Rinsho-Seishin-Yakuri (Japanese Journal of Clinical Psychopharmacology 2010 13(2): 339-355. (in Japanese).

[73] C.A. Moon, D.K. Jesinger, The effects of psychomotor performance of fluvoxamine versus mianserin in depressed patients in general practice, Br J Clin Pract 45(4) (1991) 259-62.

[74] A. Perez, J.J. Ashford, A double-blind, randomized comparison of fluvoxamine with mianserin in depressive illness, Curr Med Res Opin 12(4) (1990) 234-41.

[75] R. Suchting, V. Tirumalajaru, R. Gareeb, T. Bockmann, C. de Dios, J. Aickareth, O. Pinjari, J.C. Soares, P.J. Cowen, S. Selvaraj, Revisiting monoamine oxidase inhibitors for the treatment of depressive disorders: A systematic review and network meta-analysis, J Affect Disord 282 (2021) 1153-1160.
